# Supplementary material for: Modeling mechanochemical coupling in optogenetically activated cell layers
Source: Biophys J. 2025 Oct 9;125(12):3034–47. doi: 10.1016/j.bpj.2025.10.002 (PMC13507329; doi:10.1016/j.bpj.2025.10.002)
Supplement: Supplementary file 2 [file mmc6.pdf]

Biophysical Journal
Article

Modeling mechanochemical coupling in optogenetically activated cell layers

Dennis Wörthmüller,[1] Falko Ziebert,[2,3] and Ulrich S. Schwarz[2,3,*]
[1]Institut Curie, Universite PSL, Sorbonne Universite, CNRS UMR168, Physique des Cellules et Cancer, Paris, France; [2]Institute for Theoretical Physics, Heidelberg University, Heidelberg, Germany; and [3]BioQuant–Center for Quantitative Biology, Heidelberg University, Heidelberg, Germany

ABSTRACT In adherent cells, actomyosin contractility is regulated mainly by the RhoA signaling pathway, which can be controlled by optogenetics. To model the mechanochemical coupling in such systems, we introduce a finite element framework based on the discontinuous Galerkin method, which allows us to treat cell doublets, chains of cells, and monolayers within the same conceptual framework. While the adherent cell layer is modeled as an actively contracting viscoelastic solid on an elastic foundation, different models are considered for the Rho pathway, starting with a simple linear chain that can be solved analytically and later including direct feedback that can be solved only numerically. Our model predicts signal propagation as a function of coupling strength and viscoelastic timescales and identifies the conditions for optimal cell responses and wave propagation. In general, it provides a systematic understanding of how biochemistry and mechanics simultaneously contribute to the communication of adherent cells.

SIGNIFICANCE Biochemical circuitry and mechanical deformations are tightly coupled in adherent cells. In general, each mechanical deformation leads to changes in the concentrations of the signaling molecules; in addition, specific molecules have evolved mechanosensitivity that feeds back into the signaling pathways. In multicellular systems, both biochemical and mechanical signals are propagated across cell-cell boundaries. Here, we demonstrate that the discontinuous Galerkin finite element method is a natural and numerically highly efficient way to model the interplay between biochemistry and cell mechanics in layers of adherent cells.

INTRODUCTION

With the advent of mechanobiology, it has become clear that biochemistry and mechanics play an equally important role for the function of cellular systems (1,2). For example, it has been shown that stem cells actively sense the stiffness of their environment, and that this mechanical input together with soluble factors determines their subsequent differentiation (3,4). Later it has been shown that one important element is the transcription factor Yes-associated protein, which is activated on stiff substrates and in geometrical confinement (5,6).

A close coupling between biochemistry and mechanics also exists for multicellular systems. For example, it has been shown that in wound healing and infection, neighboring epithelial cells coordinate their activity by mechanical activation of extracellular signal-regulated kinases (ERKs) (7,8). A mathematical model demonstrated how wave propagation results from mutual feedback between mechanics and biochemistry: leader cells pull on their followers, ERK is activated in the followers, and it activates contractility, which leads to forces on the next row of followers (9–11). A similar mechanism is also realized by the tumor suppressor protein merlin, which under mechanical force is relocalized from the cell-cell junctions to the cytoplasm (12). Interestingly, the mechanism for wave propagation is similar to the one for action potentials in the neurosciences. In principle, the signal could go both ways, but a refractory period in the sending part prevents the wave from going backward.

The general scheme of mechanochemical feedback leading to complex systems behavior is even more evident for developing organisms (13,14). Here, morphogen concentration fields determine where cells grow and divide, and this leads to changes of the domain in which signaling is active, which in turn changes the way morphogens are secreted and distributed. Such feedback loops lead to nonlinear systems dynamics, which can explain the intricate patterns that

Submitted June 30, 2025, and accepted for publication October 2, 2025.
*Correspondence: schwarz@thphys.uni-heidelberg.de
Editor: Joachim Raedler.
https://doi.org/10.1016/j.bpj.2025.10.002
© 2025 The Author(s). Published by Elsevier Inc. on behalf of Biophysical Society.
This is an open access article under the CC BY license (http://creativecommons.org/licenses/by/4.0/).

emerge during tissue formation and embryogenesis (13). One prominent model organism is the fruit fly *Drosophila*, for which experimental observations have been coupled with mathematical models (15,16). Another one is the freshwater polyp *Hydra*, which is able to regenerate its patterning even after being cut in pieces (17–20). However, for such organismal systems, it is very challenging to achieve a systems level understanding connecting molecular processes to the tissue scale. For mechanistic understanding, it is therefore rewarding to turn back to their elementary building blocks, the cells, and small assemblies of such cells, with the long-term aim to upscale to larger systems.

To understand the coupling of biochemistry and mechanics at the level of single cells, one must start with the actin cytoskeleton (21–23). This is a network of actin filaments and myosin II molecular motor proteins that determine the mechanical properties of cells, particularly during processes such as adhesion, migration, and division. Since the actin cytoskeleton continuously consumes energy in the form of adenosine triphosphate to grow and reorganize its filaments, generating forces and flows in the process, the appropriate modeling approach is to introduce active stresses that are coupled to the chemical potential of the myosin II motors. This central concept led to the development of active gel theory (24,25). Active gel theory is commonly used to model single-cell migration (26,27), but it can also be readily extended to larger systems, such as tissue flow (28).

To incorporate more mechanistic details into the process of force generation by cells, one must also consider how it is regulated by the small GTPases from the Rho family, including RhoA, Rac1, and Cdc42 (23,29). Briefly, Rac1 and Cdc42 primarily regulate the assembly of larger protrusive actin structures, such as lamellipodia and filopodia, respectively, whereas RhoA is predominantly responsible for the formation of actomyosin contractility. The activity of these small GTPases is controlled by many different guanine exchange factors (GEFs). If such a GEF activates RhoA, this in turn activates diaphanous-related formin for actin polymerization and Rho-associated protein kinase (ROCK) for contractility through myosin II molecular motors (30). Together, these effects then lead to productive force generation.

Several positive and negative feedback loops exist among these components, leading to complex temporal dynamics and pattern formation (23,29). For instance, Bement et al. (31) identified an activator-inhibitor relationship between RhoA and F-actin, which results in the emergence of spiral contraction waves during cytokinesis in *Xenopus* embryonic cells. Similar surface contraction waves have been observed in starfish oocytes during maturation, modeled by the coupled reaction kinetics of actin and myosin II (32,33). In a systematic study, by combining nonlinear system dynamics and experimental data, Kamps et al. (34) developed a detailed model for the reaction kinetics of GEF, RhoA, and myosin II. This model not only demonstrates the complexity of the RhoA pathway, but also successfully explains the experimental observations of pulsatile contractions in the actin cortex, identifying cytosolic GEFH1 as a crucial parameter for the emergence of this pulsatile behavior.

Although GEFs for the small GTPases from the Rho family sometimes are activated purely by biochemical pathways, often their activation results from mechanical forces (35–37), similar to the cases of ERK (7,9,10) and merlin (12). Often, theoretical models incorporate these concepts by proposing feedback mechanisms between mechanical tension and biochemical signaling. For example, a positive biochemical-mechanical feedback loop between forces exerted on focal adhesions and RhoA signaling at these sites can explain spatial gradients in the periodic myosin-α-actinin pattern in stress fibers stimulated with calyculin A (38). Several mathematical studies combined simple models for Rho GTPase activity and cell mechanics to demonstrate that their interplay leads to complex cell behaviors (39,40). The authors showed that their proposed system can exhibit bistability, where the two states represent permanently contracted or relaxed cells and can also produce oscillatory states. Recently, Staddon et al. (41) coupled a basic activator-inhibitor reaction-diffusion (RD) system, comprising RhoA as the activator and myosin II as the inhibitor, with the mechanics of viscoelastic solids and fluids. In this model, the interplay between biochemistry, actomyosin contractility, and viscoelastic deformation leads to the emergence of propagating pulsatile contractions and topological turbulence in flows of RhoA.

In order to dissect these signaling pathways experimentally, one usually works with inhibitors, which are small chemical molecules that reduce the effect of certain components like ROCK or myosin II. However, the concomitant results are often rather qualitative in nature, and it is not always clear how well the inhibitor reaches its putative target. Recently, optogenetics has emerged as a powerful alternative, which leads to more quantitative results (42). In optogenetics, a light-sensitive construct is engineered into the cells and can then be activated with high temporal and spatial resolution. This approach has been applied in many studies, for example, to control neural activity (43), the regulation of gene expression (44,45), or even to regulate engineered metabolic pathways in cells (46), which illustrates the versatility of this method. In the field of mechanobiology, it has become an established technique to activate the Rho pathway by recruiting a GEF to the membrane (33,47–49), thus allowing for a precise spatiotemporal control of cytoskeletal dynamics of single cells (50,51) and multicellular systems (52,53).

The quantitative advances achieved by optogenetics now open the door for a more detailed mathematical modeling of the underlying processes. Different modeling frameworks have been applied before to couple biochemistry and mechanics. One attractive option is the cellular Potts model,

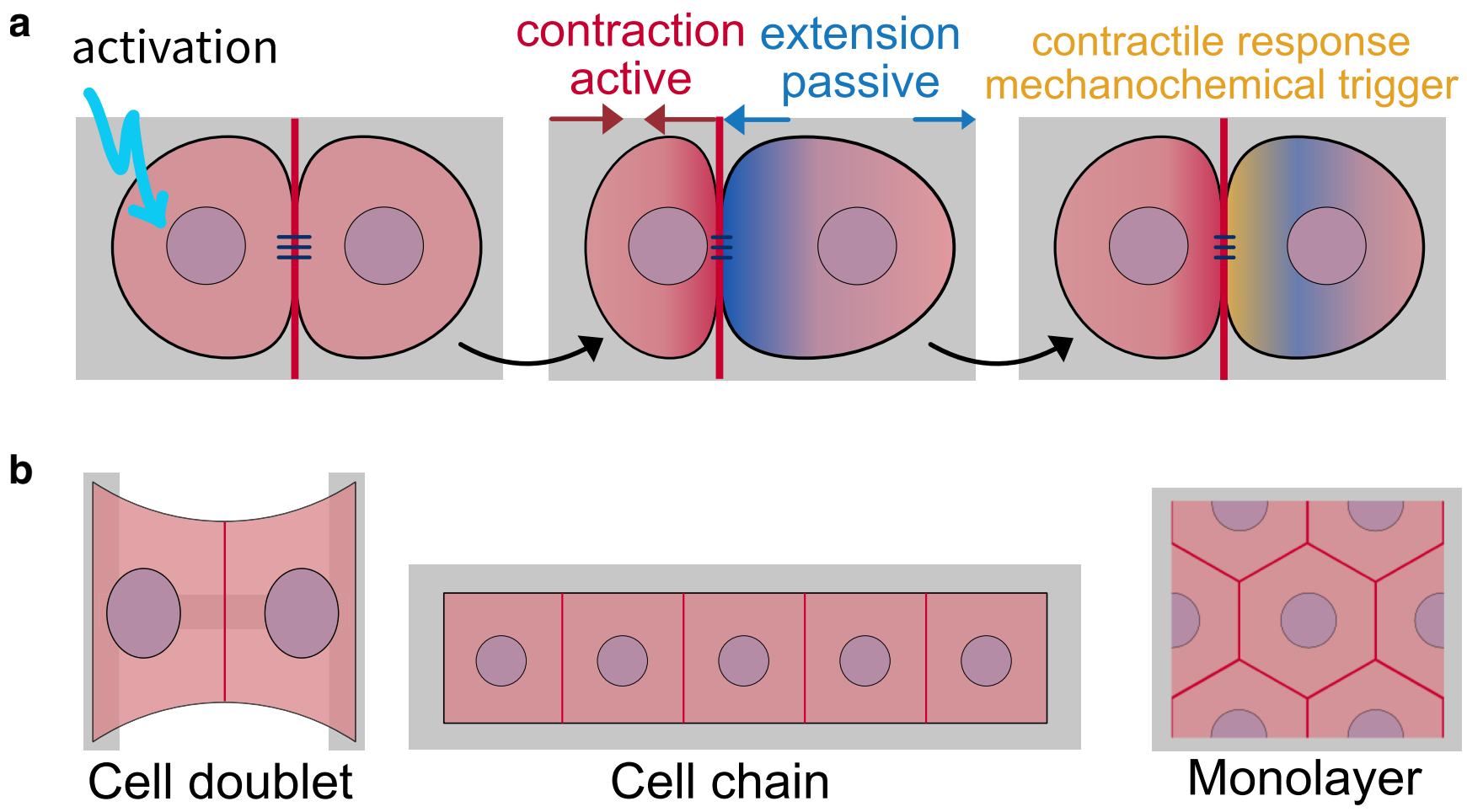

FIGURE 1 Mechanochemical coupling in different geometries. (*a*) Cells in contact with each other communicate through mechanochemical coupling. In the cell doublet, the optogenetically controlled contraction of the left cell induces an active contractile response in the right cell. (*b*) For spatial modeling, we specify adhesion geometries commonly used in experiments. We start with the cell doublet on an H pattern and then continue to cell chains and cell monolayers.

which has been applied to both Rho/Rac signaling (54) and EKR signaling (7). However, the framework is not able to model active stresses in detail. Here, we therefore turn to active continuum mechanics, which is the natural framework to describe local active stresses, as demonstrated by the success of active gel theory (24–28). For the signaling pathways, we rely on a description in terms of a system of RD equations (34) solved on a geometrical domain that represents a cell ensemble strongly adhered to an elastic foundation (50,55,56). Because both frameworks, continuum mechanics and RD systems, lead to partial differential equations (PDEs), we turn to the finite element method (FEM), which is a standard way to numerically solve PDEs. In particular, since we aim to model multicellular systems, for the RD part, we implement the discontinuous Galerkin (DG) version of FEM, which offers a natural way to represent the discontinuities in concentrations at the cell-cell boundaries in multicellular systems. Here, we first lay the conceptual basis for such an approach and then present representative applications for mechanochemical pattern formation in multicellular systems controlled by optogenetics.

The manuscript is structured as follows. First, we will introduce the main concepts and equations, motivated by recent experiments on optogenetic activation of cell doublets (52). Our starting point is the observation that optogenetic activation of contractility in one cell triggers an active response in a neighboring cell; compare Fig. 1 *a*. For Rho activation, we start with a simple linear variant of the pathway, which is sufficient to describe the recent experiments. We then explore the consequences of this response, going from the cell doublet on an H pattern to increasingly larger systems, namely cell chains and monolayers as commonly used in experiments (52,53); compare Fig. 1 *b*. Finally, we will demonstrate the generality of the simulation framework by addressing the case of a monolayer with a more dynamic model for the reaction kinetics of the Rho pathway (34).

MATERIALS AND METHODS

Coupling biochemistry and mechanics

Our modeling approach is strongly motivated by recent experiments on cell doublets on an H pattern whose contractility is activated in the left cell by Rho optogenetics, such that one can follow the response of the right cell in quantitative detail (52). The formulation of the model follows the central observation that actively generated stresses within the cell layer depend on the concentration of the downstream output of the RhoA pathway. Assuming that sufficient amounts of actin filaments are generated by the diaphanous-related formin leg, this is mainly the amount of active myosin II generated by the ROCK leg of the pathway. The spatiotemporal distribution of actively generated stresses depends also on the reaction kinetics and diffusive properties of all upstream signaling proteins. The active stresses may then lead to deformation of the cell, which directly feeds back to the RD system by generating advection terms and changing concentrations. Consequently, the spatiotemporal evolution of a signaling protein concentration $c_i(\mathbf{x},t)$ is described by a reaction-diffusion-advection equation on a two-dimensional time-dependent domain $\Omega(t)$,

$$\frac{\partial c_i}{\partial t} + \nabla \cdot (\mathbf{v}(\mathbf{x},t)c_i) = \nabla \cdot (\mathbf{D} \cdot \nabla c_i) + R_{c_i}(t), \qquad (1)$$

where $\mathbf{D}$ denotes the two-dimensional diffusion tensor, R_{c_i} the reaction kinetics, and the index i represents a signaling protein in the RhoA pathway. Equation 1 arises naturally by demanding local mass conservation on the time-dependent domain by following Reynold's transport theorem. It includes an advection term $\mathbf{v}\cdot\nabla c_i$ due to flows induced by contraction and expansion and an enrichment/dilution term $c_i\nabla\cdot\mathbf{v}$ due to local volume changes, where $\mathbf{v}(\mathbf{x},t)$ corresponds to the velocity of the deforming material. Hence, deformations naturally interfere with the spatiotemporal evolution of the protein concentrations. Equation 1 is written in terms of spatial (Eulerian) coordinates $\mathbf{x}$, which is a convenient choice for the description of diffusion processes.

However, the deformation of the cell domain is better treated in terms of referential (Lagrangian) coordinates $\widehat{\mathbf{x}}$. The two coordinate systems are related by the deformation field $\widehat{\mathbf{u}}(\widehat{\mathbf{x}},t) = \mathbf{x}(\widehat{\mathbf{x}},t) - \widehat{\mathbf{x}}$. We use Piola's identity $\widehat{J}\nabla\cdot\mathbf{a} = \widehat{\nabla}\cdot(\widehat{J}\widehat{\mathbf{F}}^{-1}\cdot\widehat{\mathbf{a}})$, where $\mathbf{a}$ is an arbitrary vector field and $\widehat{\mathbf{F}} = \partial\mathbf{x}/\partial\widehat{\mathbf{x}} = \mathbf{I} + \widehat{\nabla}\widehat{\mathbf{u}}$ the deformation gradient tensor with $\widehat{J} = \det(\widehat{\mathbf{F}})$, to pull Eq. (1) back to the reference configuration Ω_0 and express it in terms of Lagrangian coordinates as

$$\frac{\partial}{\partial t}(\widehat{J}\widehat{c}_i) - \widehat{\nabla}\cdot\left(\widehat{J}\mathbf{D}\widehat{\mathbf{C}}^{-1}\cdot\widehat{\nabla}\widehat{c}_i\right) - \widehat{J}R_{\widehat{c}_i}(t) = 0\,. \qquad (2)$$

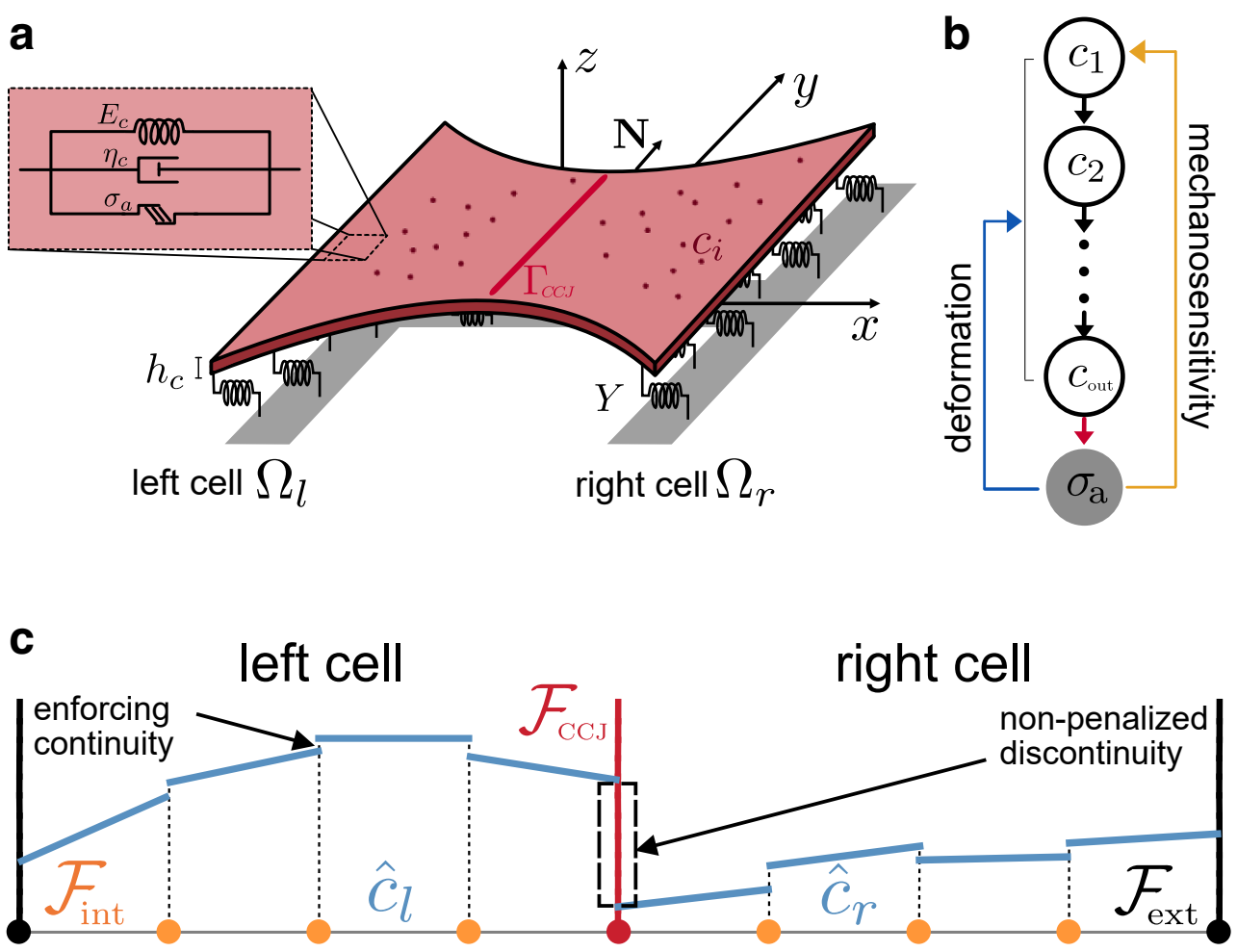

FIGURE 2 Mechanochemical model for a cell doublet. (*a*) A cell doublet (Young's modulus E_c, viscosity η_c, active stress σ_a, contractile layer thickness h_c) adheres to an elastic foundation via springs with stiffness density Y that is homogeneously distributed over the area defined by the H-shaped micropattern. The cell-cell junction (*red line*; Γ_{CCJ}) separates the two cells (Ω_l, Ω_r) along the vertical symmetry axis of the micropattern (*y-axis*). (*b*) The simplest model for the signaling cascade is a linear chain in which mechanosensitivity leads to activation of the first signaling protein, and the last signaling protein determines active stresses. (*c*) Discontinuous Galerkin method for the reaction-diffusion system. Solutions are naturally allowed to have discontinuities across the mesh facets. Penalizing discontinuities at internal facets $\mathcal{F}_{\mathrm{int}}$ (*orange dots*) leads to approximately smooth solutions within cells. Dropping this penalty at the cell-cell boundary $\mathcal{F}_{\mathrm{CCJ}}$ results in jumps.

Here, $\widehat{\nabla}$ denotes the derivative with respect to Lagrangian coordinates, $\mathbf{D} = D\mathbf{I}$ is assumed to be an isotropic tensor with scalar diffusivity D, and $\widehat{\mathbf{C}} = \widehat{\mathbf{F}}^{\mathrm{T}}\widehat{\mathbf{F}}$ denotes the Cauchy-Green deformation tensor. The velocity of the material is absorbed into the time derivative in the first term. For the details of this derivation, we refer to the supporting material. From the first term in Eq. (2), we see that compression ($\partial_t\widehat{J} < 0$) and dilation ($\partial_t\widehat{J} > 0$) of the elastic domain effectively alter the reaction kinetics. Further, we see that the diffusion is impacted by local deformations and can become anisotropic.

Following earlier work on cells as active materials, we describe the mechanics of the cell layer as a viscoelastic continuum with active stresses coupled to an elastic foundation (50,55,57,58) (compare Fig. 2 *a*). In particular, we assume that the lateral extent of the cell layer is much larger than the thickness h_c of its effective contractile layer at the basal side. This assumption also underlies the widely used method of monolayer stress microscopy (52,59–61). Note that h_c is usually not larger than 1 μm, whereas the cell height for strongly adhering cells typically varies between 2 and 10 μm, depending on cell type, cell density, and shape of the nuclei. The thin layer assumption allows us to obtain a two-dimensional model by using the plane stress conditions for the elastic sheet. The force balance equation then reads

$$\nabla \cdot \boldsymbol{\sigma} + \mathbf{f} \;=\; 0, \tag{3}$$

where $\boldsymbol{\sigma}$ is the two-dimensional in-plane Cauchy stress tensor, and $\mathbf{f}$ denotes an externally applied two-dimensional body force. The two-dimensional stress tensor is obtained by averaging the three-dimensional stress tensor over the thickness of the cell layer.

For the cell layer, we assume a linear viscoelastic constitutive relation of the solid (Kelvin-Voigt) type. Although cells are very dynamic and often are modeled by viscoelasticity of the fluid (Maxwell) type, especially in active gel theory (25), here, we consider stably adhering cells that effectively behave as solids due to homeostatic mechanisms, including volume control. The Kelvin-Voigt law reads

$$\boldsymbol{\sigma}_{\mathrm{p}} \;=\; \left(1 + \tau_c\frac{\partial}{\partial t}\right)(\lambda \mathrm{tr}(\boldsymbol{\varepsilon})\mathbf{I} + 2\mu\boldsymbol{\varepsilon}), \tag{4}$$

where $\boldsymbol{\varepsilon}=(\nabla\mathbf{u}+\nabla\mathbf{u}^{\mathrm{T}})/2$ denotes the infinitesimal strain tensor, and $\tau_c = \eta_c/E_c$ is the relaxation time defined by the ratio of viscosity η_c and Young's modulus E_c of the cell. Further, we introduce the two-dimensional Lamé coefficients as

$$\lambda \;=\; \frac{\nu_c h_c E_c}{1 - \nu_c^2}, \quad \mu \;=\; \frac{h_c E_c}{2(1+\nu_c)}, \tag{5}$$

where ν_c is the cellular Poisson's ratio.

Actomyosin contractility is modeled by an active stress tensor $\boldsymbol{\sigma}_{\mathrm{a}}$, and the total cell stress is given by the sum of the passive and active contributions, $\boldsymbol{\sigma} = \boldsymbol{\sigma}_{\mathrm{p}}+\boldsymbol{\sigma}_{\mathrm{a}}$. The cell layer is coupled to an elastic substrate, which can be thought of as a continuous layer of springs between the cell and a rigid substrate (55,62). In particular, the elastic foundation can also describe an elastic substrate as commonly used in traction force microscopy. It is described by a local force per unit area

$$\mathbf{f} \;=\; -\,Y\mathbf{u}, \tag{6}$$

where Y is the spring constant density ($[Y] = \mathrm{N\,m^{-3}}$) and $\mathbf{u}$ the displacement field of the cell layer. In case of a micropatterned surface, the spring constant density becomes position dependent and defines the adhesion geometry. Note that $\boldsymbol{\sigma}$ measures stresses in the deformed configuration and is evaluated at spatial coordinates. This means that in order to express Eq. (3) in terms of referential coordinates, one has to use the first Piola-Kirchhoff stress tensor $\widehat{\mathbf{P}}$. This distinction becomes important only in the limit of finite strains. In the limit of small strains, i.e., small deformation gradients, we can assume $|\widehat{\nabla}\widehat{u}| \ll 1$ and only consider terms up to linear order in $\widehat{\nabla}\widehat{u}$ for which we can approximate $\widehat{\mathbf{F}}^{-1}\approx\mathbf{I} - \widehat{\nabla}\widehat{u}$, $\widehat{J}\approx 1 + \mathrm{tr}(\widehat{\nabla}\widehat{u})$, and $\widehat{\mathbf{C}} = \mathbf{I} + \widehat{\nabla}\widehat{\mathbf{u}} + \widehat{\nabla}\widehat{\mathbf{u}}^{\top} + \mathcal{O}(|\widehat{\nabla}\widehat{\mathbf{u}}|^2)\approx\mathbf{I} + 2\boldsymbol{\varepsilon}$. Given this small strain assumption, the two tensors only differ in terms $\mathcal{O}(|\widehat{\nabla}\widehat{\mathbf{u}}|^2)$, such that $\boldsymbol{\sigma}\approx\widehat{\mathbf{P}}$.

The boundary of the cells is nonpermeable for the signaling proteins, and hence, we impose zero-flux boundary conditions at the interface between cell interior and cell exterior $\mathbf{j}\cdot N = 0$ on $\partial\Omega_0$ with $\mathbf{j} = \widehat{J}\mathbf{D}\widehat{\mathbf{C}}^{-1}\cdot\widehat{\nabla}\widehat{c}_i$ being the diffusive flux. Besides ensuring mechanical integrity, an inherent feature of intercellular junctions is to maintain compartmentalization in tissues by acting as a barrier for fluids and solutes. Therefore, also the cell-cell junctions (CCJs) are nonpermeable, which is incorporated by imposing an internal zero-flux boundary condition on Γ_{CCJ}. As no external stresses are applied at the boundary $\partial\Omega_0$ of the sheet, we have the boundary condition $\boldsymbol{\sigma}\cdot N = 0$ on $\partial\Omega_0$. The net traction force exerted by the cell layer vanishes as $\int_{\Omega_0} Y\mathbf{u}\;\mathrm{d}\Omega_0 \;=\; 0$, as required for a closed system.

In summary, the model can be formulated as follows: find the displacement field $\mathbf{u}$ together with the concentrations $\widehat{c}_i$ of the signaling species such that

$$\nabla \cdot \boldsymbol{\sigma} \;=\; Y\mathbf{u} \;\text{ in }\; \Omega_0, \tag{7}$$

$$\frac{\partial}{\partial t}(\widehat{J}\widehat{c}_i) \;-\; \widehat{\nabla}\cdot\left(\widehat{J}\mathbf{D}\widehat{\mathbf{C}}^{-1}\cdot\widehat{\nabla}\widehat{c}_i\right) \;-\; \widehat{J}R_{\hat{c}_i}(t) \;=\; 0 \;\text{ in }\; \Omega_0, \tag{8}$$

together with the boundary conditions

$$\boldsymbol{\sigma}\cdot\mathbf{N} \;=\; 0 \quad \text{on } \partial\Omega_0, \tag{9}$$

$$\left(\widehat{J}\mathbf{D}\widehat{\mathbf{C}}^{-1}\cdot\widehat{\nabla}\widehat{c}_i\right)\cdot\mathbf{N} \;=\; 0 \;\text{ on }\; \partial\Omega_0, \tag{10}$$

$$\left(\widehat{J}\mathbf{D}\widehat{\mathbf{C}}^{-1}\cdot\widehat{\nabla}\widehat{c}_i\right)\cdot\mathbf{N}_{\mathrm{CCJ}} = 0 \text{ on } \Gamma_{\mathrm{CCJ}}. \quad (11)$$

In addition to the geometrically arising coupling between the RD system and mechanics as described by Eqs. 7 and 8, we introduce two additional coupling mechanisms as schematically illustrated in Fig. 2 *b*. First, we couple the output of the RD system, i.e., the concentration of the last signaling protein of the activation cascade c_{out}, to active stresses σ_{a} (red arrow in Fig. 2 *b*). For this, we relate $\widehat{c}_{\mathrm{out}}(\widehat{\mathbf{x}},t)$ to σ_{a} via a relation (41,63)

$$\sigma_{\mathrm{a}}(\widehat{c}_{\mathrm{out}}) = \sigma_0 \tanh(S\,\widehat{c}_{\mathrm{out}}), \quad (12)$$

where σ_0 is the maximal contractile stress, and S a parameter that controls how sensitive stress generation is. Since $tanh(x) \leq 1$ everywhere, we additionally ensure that active stresses are bound and hence avoid numerical instabilities. Second, we introduce mechanosensitivity by relating the mechanical perturbations to the activation of the most upstream signaling protein $\widehat{c}_1$ in the RD system; see Fig. 2 *b* (yellow arrow). In our model, the mechanical perturbation can either be force related (measured in terms of internal stresses) or deformation related (expressed in terms of strain or compression/stretch). From the perspective of continuum mechanics, these measures are provided by the Cauchy stress tensor $\boldsymbol{\sigma}$ and the Cauchy strain tensor $\boldsymbol{\varepsilon}$ (or the deformation gradient tensor $\mathbf{F}$). To make this coupling independent of the frame of reference, for each of the different measures, we can choose between two tensor invariants (for a 2D system), the trace or the determinant (64). Motivated by experimental studies in a strain-controlled experimental setup, showing that cells respond directly to stretch (65), we decide to introduce a strain-dependent feedback. This choice implies that we use the trace of the strain tensor, $\mathrm{tr}(\boldsymbol{\varepsilon})$, because the determinant $det(\boldsymbol{\varepsilon})$ is at least of order ε^2 and not suitable for a linear constitutive relation. However, in more detailed models of mechanotransduction, higher order invariants could be included. In this case, one also had to go to nonlinear material laws, e.g., Neo-Hookean, for consistency. We also note that mechanosensitive proteins usually respond to extension, not to compression, and thus, we use strain only if it is extensile. Hence, we assume an activation rate of the first signaling protein due to passive strains via a source term of the form

$$\partial_t \widehat{c}_1 = a_{c_1}\mathrm{tr}^+(\boldsymbol{\varepsilon}), \quad (13)$$

where a_{c_1} is a generic activation rate, which might depend on other quantities depending on the specific choice for the RD system (similar to Hino et al. (7)). The plus sign indicates that coupling is only present in regions of positive strains, i.e., $\mathrm{tr}^+(\boldsymbol{\varepsilon}) := max(0,\mathrm{tr}(\boldsymbol{\varepsilon}))$. We want to emphasize that the magnitude of a_{c_1} has to be chosen such that if multiplied by $\mathrm{tr}^+(\boldsymbol{\varepsilon})$, it results in an appropriate rate for the strain-dependent feedback.

Discontinuous Galerkin method for reaction-diffusion system

To capture sharp concentration changes at CCJs in our RD-system. we employ a DG finite element scheme to discretize the pulled-back Eq. (8). If a standard continuous Galerkin (CG) formulation were used, the concentration field $\widehat{c}$ would be forced to remain continuous across all element boundaries (mesh facets $\mathcal{F}$), including the element boundaries representing the CCJ. However, the relevant signaling proteins do not cross cell-cell boundaries, meaning concentrations may differ significantly between neighboring cells, and fluxes across the cell-cell boundary remain zero. Using CG, this would require solving a separate system of PDEs in each cell and manually enforcing the interface conditions. By contrast, DG is a natural choice for concentration differences at cell-cell interfaces. The key idea of this method is sketched in Fig. 2 *c*. In DG the basis functions are not required to be continuous across facets. Continuity can instead be enforced in a weak sense by adding a penalty term that discourages jumps of the solution across facets. On internal facets $\mathcal{F}_{\mathrm{int}}$ (orange dots in Fig. 2 *c*), we apply this penalty to obtain an approximately continuous solution within each cell (small jumps at facets). On facets $\mathcal{F}_{\mathrm{CCJ}}$ representing the CCJs (red dots in Fig. 2 *c*), however, we relax this penalization, naturally allowing concentrations to vary between cells (large jumps at facets) while ensuring that no flux crosses the CCJ. Zero flux across external facets $\mathcal{F}_{\mathrm{ext}}$ is ensured by natural boundary conditions, Eq. (10). In practice, we use the symmetric weighted interior penalty variant (66), which is particularly well suited for problems with heterogeneous diffusion (Eq. (8)). A derivation of the weak form is provided in the supporting material. The mechanical part of our model is solved with standard CG, which is sufficient because displacements are continuous across the cell-cell boundary.

Simple model for the Rho pathway with optogenetic activation

Although the RhoA pathway allows for complex dynamics, earlier work has shown that at least in cells with strong adhesion, the actin cytoskeleton is regulated in the vicinity of a stable fixed point of this pathway (50,52). One aspect could be that strongly adherent cells exhibit dominant stress fibers. Since stress fibers are highly organized structures, it is plausible to assume a differently organized RD system than for, e.g., the homogeneous actin cortex in egg cells (31). Indeed, it has been shown recently that different organizations of the actin cytoskeleton lead to different activation and relaxation times in the Rho pathway (50). Another aspect might be the observation that the stability of the RhoA pathway has a strong dependence on the total GEF concentration (34). This suggests that the total GEF expression levels, which are naturally elevated in cells transfected with an optogentic construct, render their RhoA system more stable. Since cells transfected with the CRY2/CIBN system show a significantly higher baseline contractility, we assume that this might correspond to the stable branch of high GEF concentrations (34).

First, we want to focus on this regime and describe optogenetic activation as a reversible process such that after activation, cells eventually go back to their homeostatic contractility level without showing any significant oscillatory or excitable behavior upon photoactivation. Excitability is therefore completely controlled by the recruitment of a GEF to the membrane and can be scaled by the duration of the activation light pulse until saturation sets in (47,48). Motivated by these observations, we first assume a linear input-output relationship between GEF plasma membrane recruitment and myosin II induced contractility. This assumption not only reduces the number of unknown parameters, but it also allows for an analytical solution of the homogeneous system, such that it can be fully understood. Our proposed RhoA-myosin reaction scheme is shown in Fig. 3 *a*. GEF activity enters implicitly through a predefined input signal, which we describe by a function

$$g(t) = g_{\mathrm{ss}} + g_{\mathrm{a}}e^{-\lambda(t-t_{\mathrm{act}})}H(t-t_{\mathrm{act}}) = g_{\mathrm{ss}} + \delta g(t). \quad (14)$$

Here, g_{ss} represents a normalized steady-state GEF concentration (fraction of active GEF, for $t \leq t_{\mathrm{act}}$). After an abrupt light-mediated increase of concentration g_{a} at $t = t_{\mathrm{act}}$ (where $H(t)$ is the Heaviside function), the time course of GEF concentration for $t > t_{\mathrm{act}}$ follows a decaying exponential.

This input signal consequently triggers a reaction cascade by activating RhoA, which in turn activates myosin II. All reactions are modeled by a law of mass action with positive valued activation rate constants a and $\tilde{k}$.

Further, we assume that all active components deactivate spontaneously described by the positive valued rate constants b and s, and we express the reaction kinetics as

$$\frac{\mathrm{d}R}{\mathrm{d}t} = ag(t)(R_T - R) - bR, \quad (15)$$

$$\frac{\mathrm{d}M}{\mathrm{d}t} = \tilde{k}R(M_T - M) - sM, \quad (16)$$

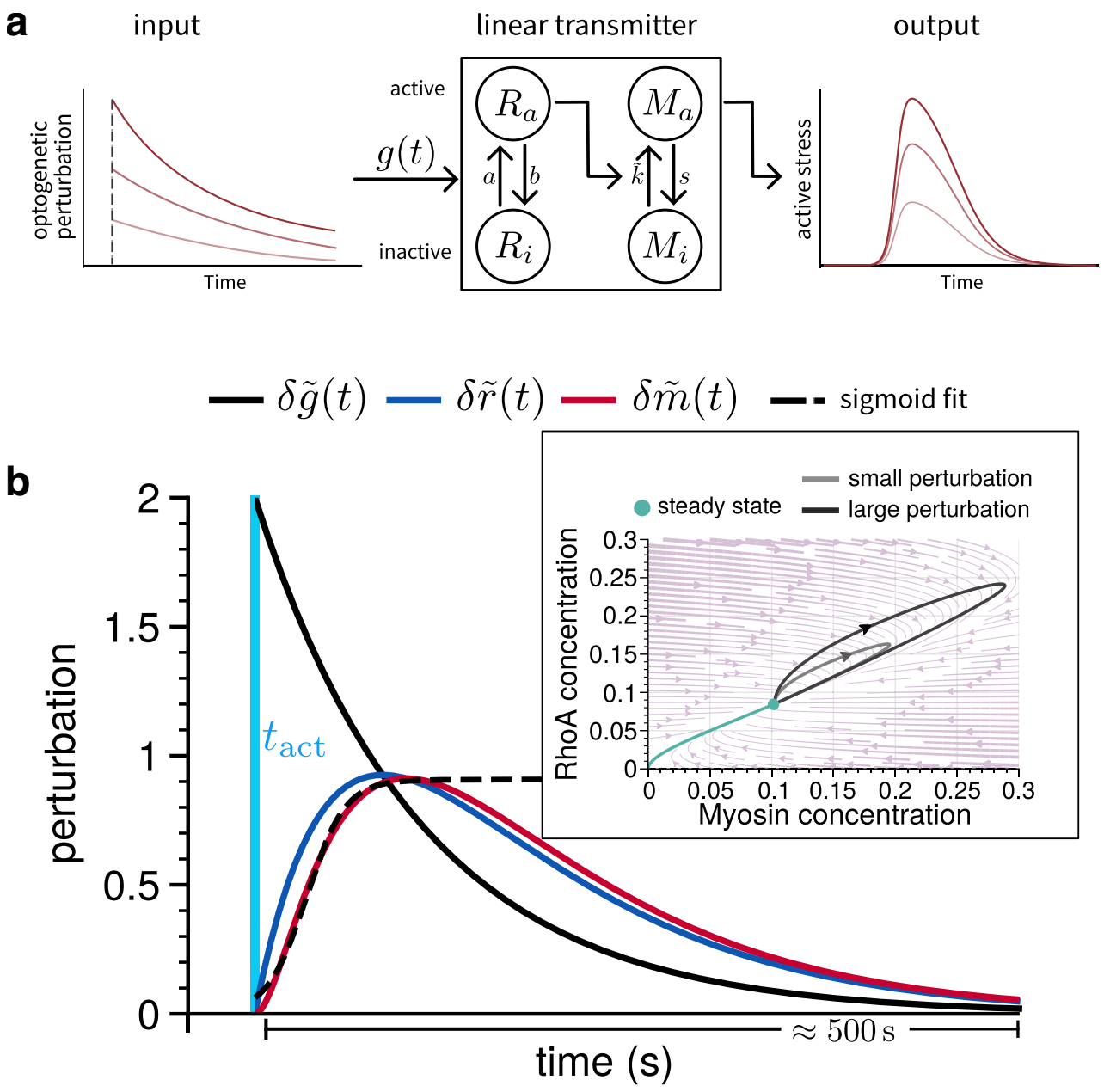

FIGURE 3 Simple model for the RhoA pathway. (*a*) Optogenetic activation of strongly adherent cells often results in a homeostatic response, which is described best by a weakly activated linear signaling cascade. For weak perturbations, the strength of the output signal scales linearly with strength of the input signal. (*b*) Time course of the normalized concentration perturbations of active RhoA (*blue*) and myosin (*red*) after rapid increase of GEF concentration (*black*) upon photoactivation at $t = t_{act}$. The dashed line represents a sigmoidal fit to the increasing edge of the myosin concentration in order to estimate the timescale of the increase $\tau_{\delta\tilde{m}} \approx 11$ s. The inset shows the time evolution of the full system of ODEs in the phase plane. The green line displays the evolution into the only stable fixed point of the system. Two perturbations for $\alpha = 2$ (*gray*) and $\alpha = 4$ (*black*) are shown and demonstrate the linearity and scalability of the system.

where for brevity, we write $R_a \equiv R$ and $M_a \equiv M$. Here, we additionally assume that the total amount of each signaling component is conserved on the studied timescale, such that concentrations of the inactive species R_i and M_i are given by the difference of the total concentration and the active concentration $R_i = R_T - R$ and $M_i = M_T - M$. Another simplification is made by considering the limit of a weakly activated signaling cascade (67) for which $R_T - R \approx R_T$ and $M_T - M \approx M_T$ such that the system can be written as

$$\frac{\mathrm{d}r}{\mathrm{d}t} = ag(t) - br, \quad \frac{\mathrm{d}m}{\mathrm{d}t} = kr - sm, \tag{17}$$

where we divided by the total concentration and hence set $r = R/R_T$, $m = M/M_T$, and $k = \tilde{k}R_T$. Before photoactivation ($t \leq t_{act}$), we have $\delta g(t) = 0$ and in this case obtain the steady-state concentrations for RhoA and myosin as

$$r_{ss} = \frac{ag_{ss}}{b}, \quad m_{ss} = \frac{ak}{bs}g_{ss}\,. \tag{18}$$

The time evolution after perturbation ($t > t_{act}$) may generally be written as

$$r(t) = r_{ss} + \delta r(t), \quad m(t) = m_{ss} + \delta m(t), \tag{19}$$

where $\delta r(t)$ and $\delta m(t)$ denote the time-dependent perturbations of the steady state. Together with Eq. (17), we end up with the time evolution of the perturbation, which is given by

$$\frac{\mathrm{d}\delta r}{\mathrm{d}t} = a\delta g(t) - b\delta r, \quad \frac{\mathrm{d}\delta m}{\mathrm{d}t} = k\delta r - s\delta m\,. \tag{20}$$

In experiments, one usually quantifies the relative activity increase with respect to the activity baseline. We therefore normalize the perturbation with respect to the steady-state concentrations and obtain

$$\frac{\mathrm{d}\delta\tilde{r}}{\mathrm{d}t} = b(\delta\tilde{g}(t) - \delta\tilde{r}), \quad \frac{\mathrm{d}\delta\tilde{m}}{\mathrm{d}t} = s(\delta\tilde{r} - \delta\tilde{m}), \tag{21}$$

with $\delta\tilde{g}(t) = \delta g(t)/g_{ss}$. The strength and time course of the relative RhoA and myosin perturbations are controlled by the two deactivation rates b and s as well as the strength of the input signal $\alpha \equiv g_a/g_{ss}$ and its decay rate λ. For the parametrization of this linearized model, we refer the reader to the supporting material. This system of equations, Eq. (21), can be solved analytically for a spatially homogeneous system (solution given in supporting material). Typical time courses of the perturbations are shown in Fig. 3 *b*, where the inset displays the time evolution of Eq. (17).

RESULTS

Optogenetic activation of a cell doublet

We start by simulating the cell doublet (cf. Fig. 2) with linear Rho signaling as shown in Fig. 3. The corresponding computer code is documented in the supporting material, and the mechanical equations are parametrized according to Table S3. We keep all parameters fixed, except the strength of the strain-dependent feedback $a_{\delta\tilde{g}}$ and the viscoelastic timescale τ_c. Since we are mainly interested in the response to the perturbation, we omit baseline contractility; i.e., we do not consider a strain in the cell layer before activation. The reference shape was chosen to resemble the typical reference shape of a maturely adherent cell doublet with a vertically oriented cell-cell junction across the symmetry center of the pattern and two pronounced invaginated arcs spanning between the vertical bars of the H-shaped micropattern (52). The cells are assumed to contract isotropically with $\boldsymbol{\sigma}_a = \sigma_a(\delta\tilde{m})\mathbf{I}$.

We optogenetically activate the left cell Ω_l at time $t = t_{act}$; compare Fig. 4 *a*. The time evolution of the GEF perturbation for $t \geq t_{act}$ is given by

$$\frac{\mathrm{d}\delta\tilde{g}}{\mathrm{d}t} = -\lambda\delta\tilde{g} + a_{\delta\tilde{g}}\mathrm{tr}^{+}(\boldsymbol{\varepsilon})1_{\Omega_r}(x), \tag{22}$$

where we include the strain-dependent feedback in the right cell via the indicator function $1_{\Omega_r}(x)$ with $1_{\Omega_r}(x) = 1$ if $x \in \Omega_r$ and $1_{\Omega_r}(x) = 0$ otherwise. This means that the strain-dependent feedback is only active in the right cell Ω_r. Having a feedback mechanism in the left cell, we can expect nontrivial temporal behavior if positive passive strains build up in the left cell.

The optogenetic activation at $t_{act} = 5$ s is achieved by setting $\delta\tilde{g}(t_{act}) = \alpha$. This time point is shown in Fig. 4 *a* with a 100% increase ($\alpha = 2$) of the steady-state GEF concentration upon photoactivation. This GEF perturbation triggers the RhoA pathway, which leads to active contraction in the activated left cell. As contraction in the left cell progresses, passive positive strains are generated in the right cell as it is stretched (Fig. 4 *b*). This stretch leads to activation of GEF and hence triggers a contractile response in the right cell. Fig. 4 *c* and *d* show the active myosin in both cells

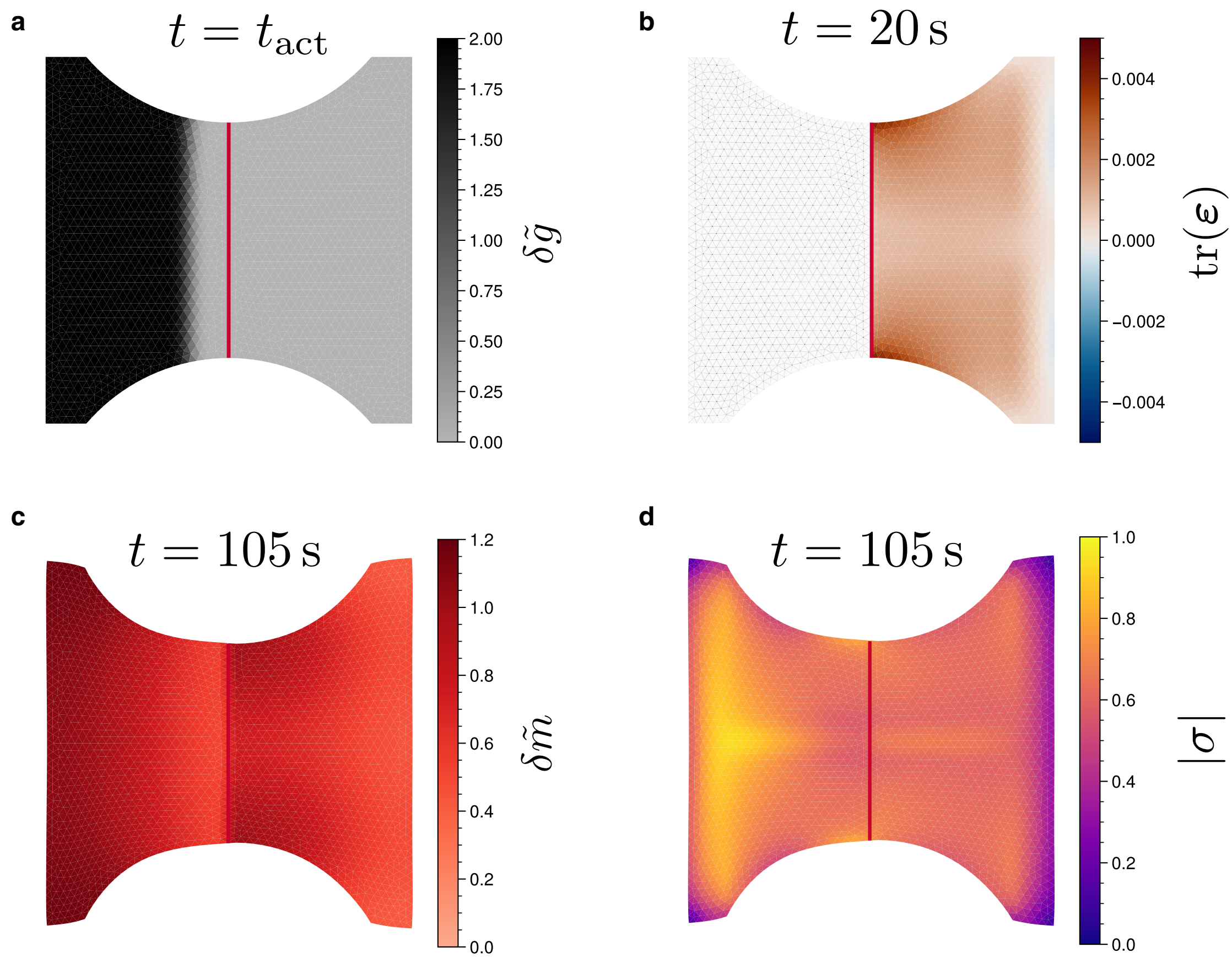

FIGURE 4 Simulation of the optogenetic activation of a cell doublet in the case of strong coupling. (*a*) Activated region (*in black*) and the GEF perturbation $\delta\tilde{g}(t_{act}) = 2$ at $t_{act} = 5$ s. In accordance with experiments, only a fraction of the left cell is illuminated in order to avoid illumination of the right cell. (*b*) Coupling measure $\mathrm{tr}(\boldsymbol{\varepsilon})$ in the nonactivated cell (*right cell*), which reaches its maximal value of approx. 5×10^{-3} after 20 s. (*c*) Myosin concentration $\delta\tilde{m}$ around the time of maximal strain energy. (*d*) Frobenius norm of the resulting Cauchy stress. The parameters for this simulation can be found in Tables S2 and S3. Further, we used $a_{\delta\tilde{g}} = 100\ \mathrm{s}^{-1}$, $\tau_c = 10$ s, $S = 1$, and time step $\Delta t = 0.5$ s. Full time sequence is shown in Video S1. Weak coupling is shown in Video S2.

as well as the Frobenius norm of the resulting Cauchy stress $\|\sigma\|_F = \sqrt{\sum_{i,j=1}^{2} |\sigma_{ij}|^2}$, respectively, for a case where parameters are chosen such that both cells deform approximately symmetrically. We note that the active myosin pattern closely follows the pattern of passive strains in the right cell with concentration peaks near the cell periphery at the cell-cell junction. Looking at the stress pattern, we further notice that the left cell contracts more strongly than the right cell. However, the invaginated arc remains fairly symmetrical. This demonstrates that a visually symmetrical contraction can occur even when both cells do not contract equally strongly, but at different locations within the cell. Here the left cell generates active stresses mainly near the vertical bar of the H pattern, whereas the right cell generates active stresses more near the periphery.

To investigate these observations further, we define different measures in order to understand the input-output relation between the left and right cell and to quantify the efficiency of the coupling. One way to quantify this is to measure the strain energy U_s transferred to the substrate,

$$U_{s,i} = \frac{1}{2}\int_{\Omega_{0,i}} Y(x)\mathbf{u}^2 \mathrm{d}\Omega_{0,i}, \quad i \in \{l, r\}, \tag{23}$$

and compare the strain energy deposited on the left and right parts of the pattern (Fig. 5 *a* (left)). Another way is to measure the asymmetry A of the contraction by quantifying the shape of the contour $y(x)$ with respect to the symmetry axis of the pattern and the inward displacement of the cell contour (Fig. 5 *a* (right))

$$A = \frac{I_l - I_r}{I}, \tag{24}$$

with $I_i = \int_{\Omega_{x,i}} y(x)\mathrm{d}x,\ i \in \{l, r\}$.

In Fig. 5 *b*, we show the shape of the invaginated arc throughout the whole contraction process. This demonstrates that the symmetry of the contraction is strongly shaped by the coupling described by the rate $a_{\delta\tilde{g}}$. For a stronger coupling (large value of $a_{\delta\tilde{g}}$) the cell doublet contracts

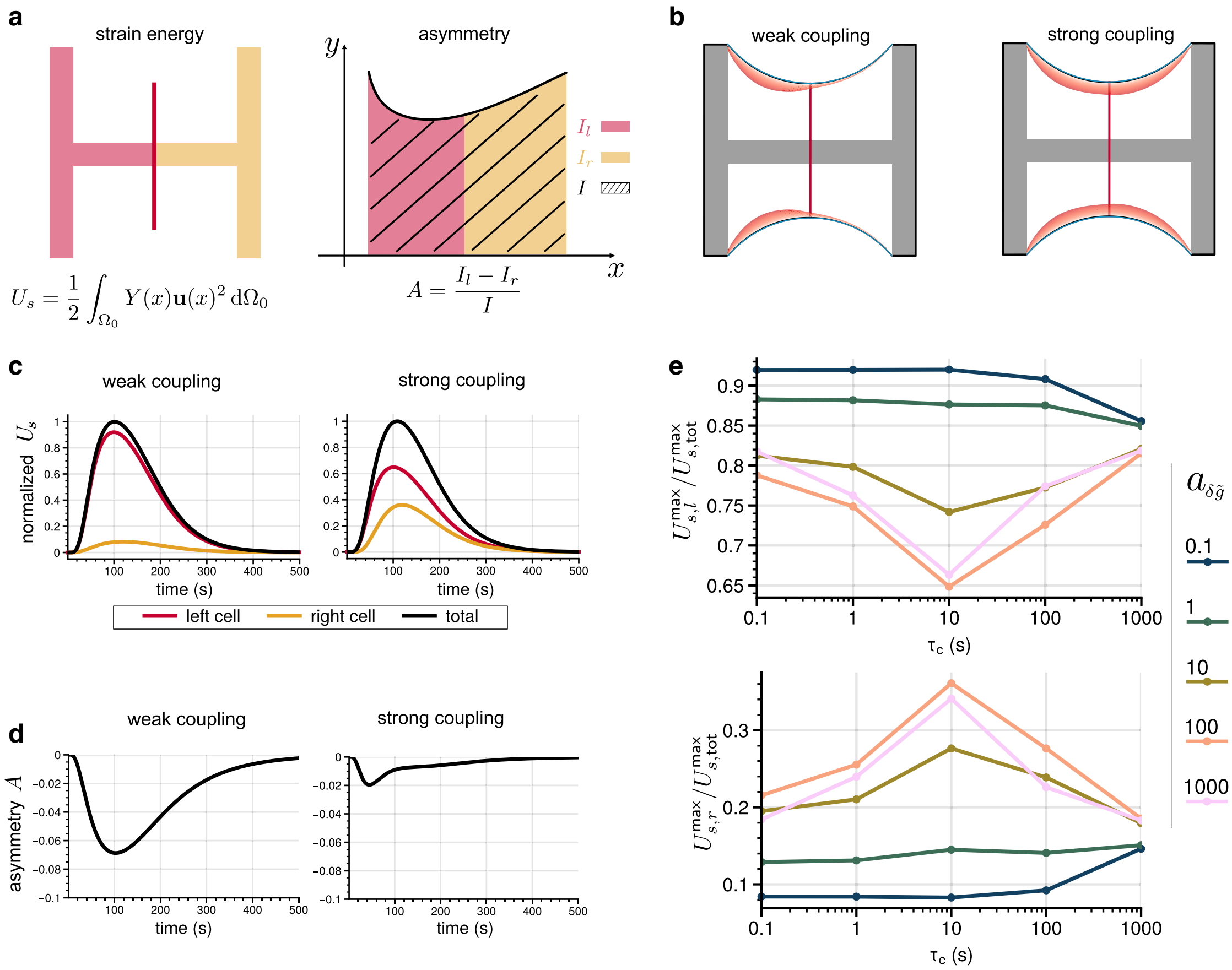

FIGURE 5 Mechanochemical coupling in cell doublets. (*a*) One measure for the asymmetry of the process is monitoring the deposited strain energies U_s in the left and right halves of the micropattern, respectively. Another measure is monitoring shape changes through asymmetry A, which follows from the integrals under the left and right halves of the contour. (*b*) Cell shapes in simulations with weak and strong active coupling. Stronger coupling may lead to visually symmetric contraction. Black line represents contour shape at the last time step. (*c*) Effects of weak and strong coupling in terms of strain energy as a function of time. Strain energy curves were normalized with respect to the maximum of the total strain energy curve. (*d*) Same for shape asymmetry. (*e*) Efficiency of the coupling defined by the ratio of the relative maxima $U^{max}_{s,l}/U^{max}_{s,tot}$ for the left versus $U^{max}_{r,l}/U^{max}_{s,tot}$ for the right cell, for different combinations of the free parameters, namely coupling strength $a_{\delta\tilde{g}}$ and viscoelastic timescale τ_c. For sufficiently large coupling, one sees optimal coupling if the viscoelastic timescale corresponds to the timescale of the linear activation cascade.

visually symmetrically in comparison to a weaker coupling (small value of $a_{\delta\tilde{g}}$), where invagination of the contour is clearly asymmetric and tends toward the activated cell.

In Fig. 5 *c*, we show the normalized strain energy U_s as a function of time, where each strain energy is normalized with respect to the maximal total strain energy $U_{s,\mathrm{tot}}$. We observe that the time course of the total strain energy is only slightly changed by the coupling strength $a_{\delta\tilde{g}}$. In the case of weak coupling, more than 90% of the deposited strain energy is generated by the left cell, and the right cell remains almost completely passive. Note that even for $a_{\delta\tilde{g}} = 0$ the strain energy on the right-hand side of the pattern is expected to be nonzero due to passive deformations by pulling of the left cell. In case of a strong coupling the right cell shows an active response and significantly contributes to the overall generated strain energy. These observations are confirmed by a quantification of the contour deformation as a function of time. For the weak coupling the contour remains strongly asymmetric throughout the majority of the simulation time. For the strong coupling, asymmetry is only present for small times until the right cell actively pulls back and symmetrizes the periphery (Fig. 5 *d*).

Until now, we only altered the rate $a_{\delta\tilde{g}}$ and kept the viscoelastic timescale τ_c constant. We next addressed the question of how the viscoelastic properties of the cell influence the efficiency of the coupling and therefore simulated several combinations of $a_{\delta\tilde{g}}$ and τ_c by varying both parameters over several orders of magnitudes. We consider the coupling to be efficient if the ratio $U^{max}_{r,l}/U^{max}_{s,tot}$ is high. The results are displayed in Fig. 5 *e*.

We find that coupling efficiency is positively correlated with the coupling strength $a_{\delta\tilde{g}}$, provided the viscoelastic timescale is such that stresses do relax on a comparable timescale. As a function of the viscoelastic timescale, we notice that coupling efficiency increases with an increasing

viscoelastic timescale, reaches an optimum around intermediate values, and decreases again for larger values of τ_c. The optimal value for τ_c is found to be at $\tau_c = 10$ s, which is in the same order of magnitude as experimentally measured values (50,68). A sigmoidal fit to the time course of the myosin concentration (Fig. 3 *b*) yields a timescale of $\tau_{\delta\tilde{m}} \approx 11$ s similar to the viscoelastic timescale. This result suggests that for an efficient mechanochemical coupling the viscoelastic timescale, i.e., the timescale at which the cell can react to mechanical stimuli, has to be in tune with the timescale at which the full linear cascade can react to those. In case of very large τ_c the left cell cannot deform sufficiently to trigger a response in the right cell. For small values of τ_c the right cell starts to counteract deformations quickly and does not remain in a stretched state for a sufficiently long time to allow the coupling mechanism to unfold its action.

Optogenetically stimulated contraction wave in a chain of cells

Having established a framework for the mechanochemical interplay of two cells, we can generalize to more cells, for example a chain of cells on a micropatterned line (compare Fig. 1 *b*). It is the strength of our DG approach that now such simulations are easy to implement and very efficient. The cell chain is realized computationally by connecting equally sized cells along the x-axis. Each cell now adheres in a homogeneous fashion to an elastic substrate. In contrast to the doublet, we here include a mechanochemical coupling also for the activated cell, so we allow for the possibility that a wave is reflected at the right and comes back to the left. Like for the cell doublet, we again assume that due to strong cell-matrix adhesion, the Rho system has a stable fixed point and that the simple model is sufficient. To model that the cells tend to polarize when placed on lines, we use a unidirectional active stress tensor such that cells only contract in longitudinal direction of the line of cells $\boldsymbol{\sigma}_a = \sigma_a(\delta\tilde{m})(\mathbf{e}_x \otimes \mathbf{e}_x)$. All parameters used in the simulation are given in Tables S2 and S4.

We now activate the left cell at $t = t_{\text{act}}$. The time evolution of the GEF concentration for $t > t_{\text{act}}$ is then described by

$$\frac{\mathrm{d}\delta\tilde{g}}{\mathrm{d}t} = -\lambda\delta\tilde{g} + a_{\delta\tilde{g}}\mathrm{tr}^{+}(\boldsymbol{\varepsilon}) \,. \quad (25)$$

As for the previous study of the cell doublet, we again vary the parameters τ_c and $a_{\delta\tilde{g}}$ to investigate the response of the system. Depending on parameters, we now observe three different responses: (1) nontransmissive (the stimulus dies out); (2) transmissive wave propagation, i.e., the most right cell is activated once; and (3) oscillatory waves going persistently through the system.

Fig. 6 *a* depicts the displacement field for the different states. Note that the displacement field is directly correlated with the generated traction forces through $\mathbf{T} = Y\mathbf{u}$. In the case of a nontransmissive parameter regime, i.e., for small $a_{\delta\tilde{g}}$, the contraction signal strongly decays during propagation from cell 1 (left) to cell 5 (right), and no substantial traction forces are generated at the right end of the line of cells; see Fig. 6 *a* on the left. This observation has been quantified by comparing the substrate strain energies generated by cell 1 and 5 as a function of time; see Fig. 6 *b* on the left.

With increasing values of $a_{\delta\tilde{g}}$, the transmission of the signal becomes more and more efficient and the active response of cell 5 comparable to cell 1; see the middle panels of Fig. 6 *a* and *b*. Since there is no absolute definition of effective transmission, a threshold criterion must be chosen, and we define the system as transmissive if $U_s^{\max,5} > 0.1 U_s^{\max,1}$.

Finally, varying also the viscoelastic timescale of the system, we observe persistent oscillatory states in the parameter regime of sufficiently large $a_{\delta\tilde{g}}$ and τ_c; see the right panels of Fig. 6 *a* and *b*. The frequency of the oscillations depends strongly on the combination of $a_{\delta\tilde{g}}$ and τ_c and can be further classified into burst-like oscillations and contractile oscillations. The former corresponds to large variations in strain energy, whereas the latter relates to small fluctuations around an elevated increased strain energy plateau (see Fig. S2).

Fig. 6 *c* shows the phase diagram as a function of the coupling strength and the viscoelastic timescale. For a strongly coupled system (i.e., sufficiently large $a_{\delta\tilde{g}}$), the transition between transmissive and oscillatory states occurs around $\tau_c \approx 10$ s, which again coincides with the timescale of the myosin relaxation. The number of cells in the line is also relevant for the signal transmission. Fig. 6 *d* shows that the transition from nontransmissive to transmissive mechanochemical signaling needs larger values of $a_{\delta\tilde{g}}$ when increasing the length of the cell chain. This emphasizes that mechanochemical signaling tends to be of dissipative nature, and hence, a cell can reach out only to a limited number of other cells, except if a strong amplification exists, like in action potentials.

Cell monolayer with nonlinear Rho pathway

We finally turn to a cell monolayer (compare Fig. 1 *b*). Again the numerical implementation is relatively easy given our DG approach, and we demonstrate this here for 28 hexagonally arranged cells. In contrast to the doublet and the line of cells, cell-matrix adhesion is now less relevant, and we do not assume that the cells in the layer are polarized in the lateral direction. Moreover, in the two-dimensional case, cells have many neighbors and therefore many inputs. These aspects suggest that the simple linear chain model for the Rho pathway might not be sufficient anymore and that more complex dynamics might arise, similar to the excitable dynamics

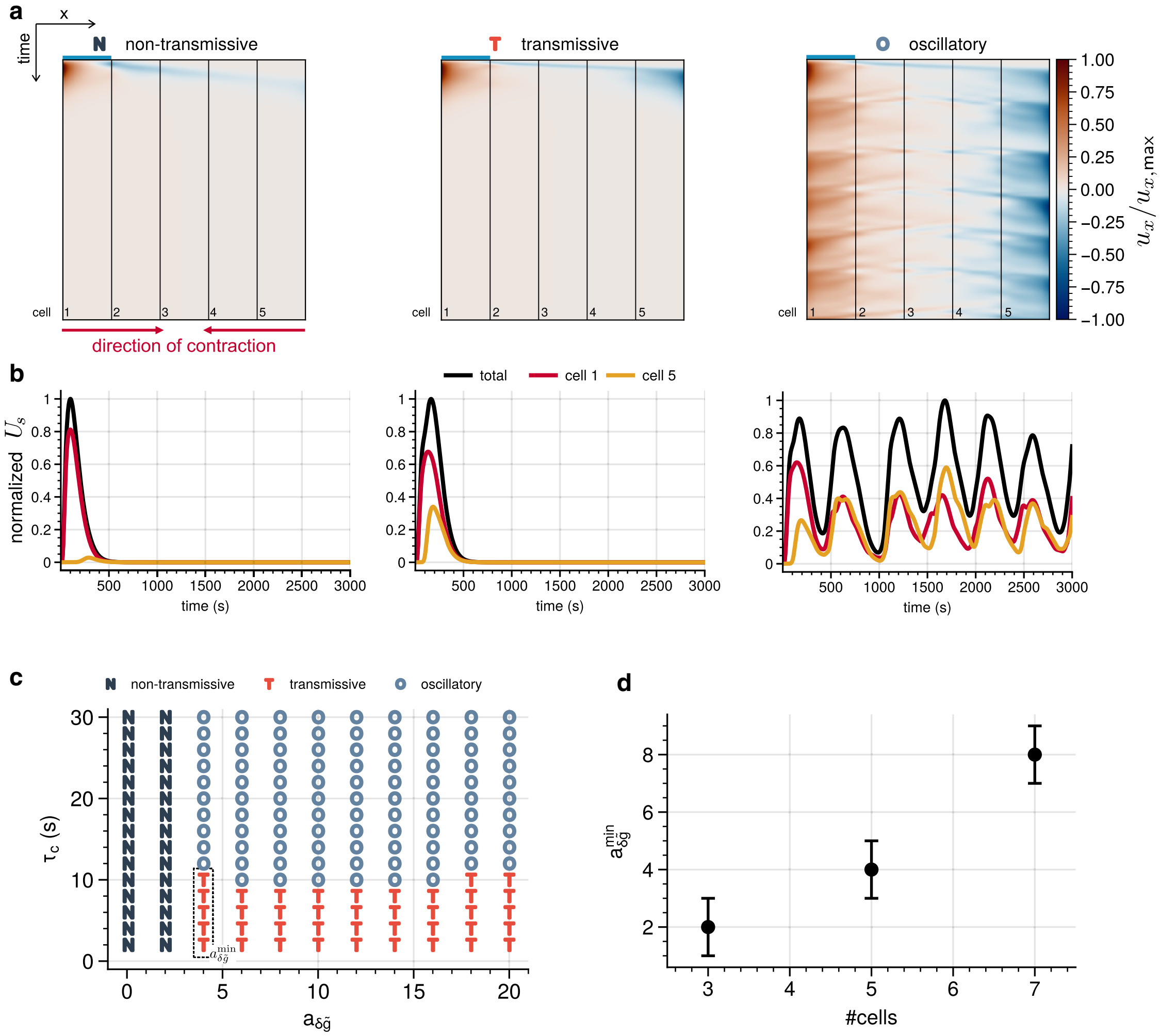

FIGURE 6 Mechanochemical coupling in a chain of cells. (*a*) Kymographs of the substrate displacement field normalized to the maximal displacement for five cells in a row and for three different conditions. At $t = 5$ s, cell 1 (*left*) is activated (*blue bar*). The contraction of the activated cell initiates a contraction wave propagating from cell 1 (*left*) to cell 5 (*right*). Depending on the parameters, we observe nontransmissive (N), transmissive (T), or oscillatory states (O). For sufficiently large strain-dependent feedback $a_{\delta\tilde{g}}$ and sufficiently large viscoelastic timescale τ_c, self-sustained oscillations emerge. (*b*) Time evolution of the strain energy for the activated cell 1 and cell 5, i.e., the last cell of the line, corresponding to the kymographs given in (*a*). (*c*) Phase diagram as a function of activation strength and viscoelastic timescale. Strong coupling and large viscoelastic times are required for oscillations. (*d*) Effect of cell number. The transition from nontransmissive to transmissive occurs at larger values of $a_{\delta\tilde{g}}$ with increasing length of the cell chain (error bars highlight discrete parameter sampling). A full simulation is given in Video S3.

known from the cortex of oocytes (31,33). We therefore now use the full nonlinear model for the Rho pathway as established earlier by Kamps et al. (34). The corresponding kinetic equations are given in Eqs. S29–S31 (supporting material). As investigated in Kamps et al. (34), they exhibit Turing-type instabilities resulting from fast (inactive) and slow (active) diffusing species paired with positive and negative feedback loops of activator-inhibitor type. Here we have chosen the parameters for the RD system such that traveling waves can form.

The cell monolayer is shown in Fig. 7 *a* with intercellular junctions shown in red and the color code depicting the levels of active stress/active myosin. The cell in the middle on the left-hand side of the monolayer was activated by inducing an instability through small random fluctuations in the GEF and RhoA concentrations (the same effect can be obtained by optogenetic activation, but for the nonlinear model, the exact mode of activation is less relevant). Several distinct contraction peaks form, and the induced deformations slowly activate the RhoA pathway and allow the active stresses to spread through the tissue, as indicated by the white arrows in Fig. 7 *a*. The initially weak and rather uniform contractions eventually turn into strong and more localized traveling contraction waves (see Fig. 7 *b*) until the whole monolayer is strongly activated; see Fig. 7 *c*. For longer times, a dynamic steady state is reached, where the deformations are most prominent near the free edges; see Fig. 7 *d*.

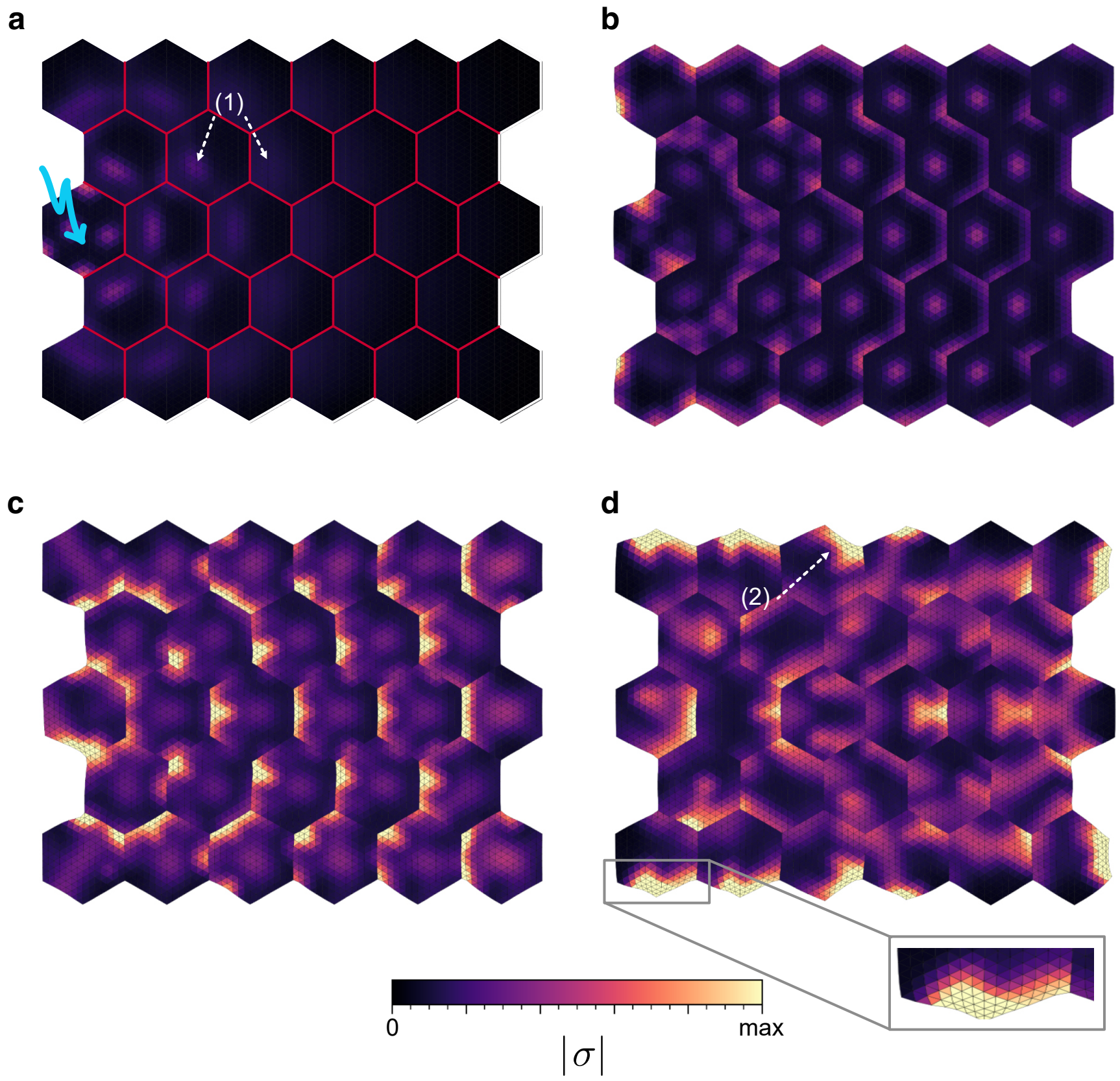

FIGURE 7 Mechanochemical coupling in a cell monolayer with a nonlinear model for the Rho pathway. (*a*) Total stress in the tissue shortly after inducing a spatiotemporal contraction pattern in the centered cell on the left side of the tissue. This initial perturbation spreads by means of the mechanochemical feedback through the tissue (*white arrows*). (*b* and *c*) Tissue at a later stage, where the initially small perturbations have developed into substantial contraction waves in each of the cells. (*d*) After transient dynamics, prominent deformations are visible in the free edges of the cell layer (*white arrow and inset*). Parameters can be found in Tables S1 and S5. Full dynamics is shown in Video S4.

DISCUSSION

Here, we have shown that the DG FEM is ideally suited to model mechanochemical coupling in cell layers. Because both the RD and the continuum mechanical models lead to PDEs, finite elements are the natural approach to couple them in a numerically efficient framework. The DG method, in addition, is ideal to describe the effect of concentration jumps at the cell-cell boundaries while still keeping the continuous nature of the PDEs for the RD system. In contrast, the PDEs for the mechanics can be treated well with standard CG, because displacements are continuous across the boundaries. Our approach is very general both in terms of biochemistry (as exemplified by the linear regulation cascade versus the fully nonlinear RD system) and the material law (here a linear Kelvin-Voigt-type material). Motivated by the homeostatic nature of adherent cell layers, it is however centered on elastic systems. For flowing systems, which are typically modeled with active gel theory (24–28), one has to switch from the Lagrangian to the Eulerian framework.

To couple the RD and mechanical subsystems, we used very simple but reasonable assumptions. Like in active gel theory, active stress is coupled to the concentration of myosin II (the last molecule of the signaling cascade), and the activation rate at the beginning of the signaling cascade (typically an exchange factor that can be controlled by optogenetics) is assumed to be proportional to the positive part of the trace of the strain tensor. In future work, one could also consider nonlinear model versions both for the material law (e.g., Neo-Hookean) and the coupling (e.g., higher order invariants of the strain tensor).

We benchmarked our approach by investigating in detail the optogenetic activation of a cell doublet on an H-shaped micropattern, as recently studied experimentally in Ref. (52). Because that work did not include any experiments on signal transduction, we cannot compare our results directly to this work. However, our work shows that the detailed RD model used here can explain the experimental observation that mechanical interactions between an optogenetically activated cell and a neighboring nonactivated cell can trigger an active contractile response. Interestingly, our simulations further predict that a cell doublet can exhibit an apparently symmetric contraction even when the left and right cells display distinct internal active stress distributions. This also shows that shape is not a unique readout of the internal state of the cell and that care has to be applied when estimating cell forces from shape.

We then exemplified the scalability of our DG approach to larger systems by looking at force and signal transmission via contraction waves in chains of cells, similar to recent experiments on optogenetic activation of cell trains (53). In these two cases, cell-matrix adhesion is very strong, and

cells tend to be homeostatic, which means that they return to baseline after optogenetic stimulation (50). Therefore, we again used the simple model of a linear chain for the Rho signaling pathway. Again a direct comparison to the experimental data is not possible because the signaling part was not investigated, but on a qualitative level, we find similar results, in particular traction force patterns with local reversals in force direction near the center of the cell train and that larger trains attenuate signal propagation.

We finally addressed the case of mechanochemically excitable monolayers, which are known to develop more complex dynamic patterns, possibly because cell-cell adhesion dominates and cells are less polarized. In particular, experiments have found that wave propagation is rather common in such systems (7,9,10), and recent simulations have shown that this should also apply to three dimensions (11). In this case, we therefore used a more comprehensive and nonlinear model for the Rho pathway (34). We then observed excitable dynamics that does not die down again, similar to experimental observations in the cortex of oocytes (31,33), but also adherent cells with intermediate activation of the Rho pathway (34).

In all of these cases, we identified conditions under which mechanochemical signaling leads to strong propagation of the signal from one cell to the other, and even to wave propagation. We find that force transmission is best when the viscoelastic timescale of the cell and the timescale of relaxation of the Rho pathway after activation are of the same order. This finding makes sense because for the bidirectional feedback to work, both processes need to work on similar timescales. The numerical value of 10 s also makes sense because this is the timescale on which adhesion contacts can remodel. We note that when elastic effects have to be faster, e.g., in the venus trap or in the mechanochemical cycle of molecular motors, the system tends to separate the slow buildup of elastic energy and its fast release. Here, however, such a separation is not needed and in fact would be a disadvantage. As shown here, the mechanochemical coupling between neighboring cells can lead to long-ranged and even wave-like signal propagation, as e.g., recently measured by optogenetic activation of the developing zebrafish neural rod (69). This agrees with earlier work on optogenetic activation in *Drosophila*, which showed that local activation can affect global processes like gastrulation (70,71).

For the cell doublet, we used the coupling to an elastic foundation as a readout of the mechanical coupling of the two neighboring cells, in addition to the asymmetry in shape. In the future, the coupling to the elastic foundation could be replaced by a continuum substrate; then one could also model the effect of mechanical cell-cell communication through the substrate, which would be a natural extension of our approach. On the biochemical side, it would be interesting to go beyond modeling of the Rho pathway and also include the effect of other known signaling molecules, including Rac/Cdc42, ERK, and merlin. The numerical DG FEM framework established here now opens the door to quickly explore the effect of such pathways and thus to establish a multiscale modeling framework that connects molecular processes to effective systems behavior.

DATA AND CODE AVAILABILITY

The Python code for the examples described above is publicly available at https://github.com/dworthmuller/MechanochemicalCoupling.

ACKNOWLEDGMENTS

U.S.S. wishes to express his gratitude to Erich Sackmann for many inspiring discussions on the physics of cells. D.W. and U.S.S. acknowledge funding through the DFG (Deutsche Forschungsgemeinschaft) grant MechanoSwitch SCHW 834/2-1. F.Z. and U.S.S. were supported by the cluster of excellence STRUCTURES funded by the DFG (EXC 2181/1–390900948). D.W. received funding from a European Research Council (ERC) grant ERC-SyG 101071793, awarded to Pierre Sens.

AUTHOR CONTRIBUTIONS

All authors designed the research together. D.W. performed the research. F.Z. and U.S.S. supervised the project. D.W. and U.S.S. wrote the original draft of the paper. All authors reviewed and approved the paper.

DECLARATION OF INTERESTS

The authors declare no competing interests.

SUPPORTING MATERIAL

Supporting material can be found online at https://doi.org/10.1016/j.bpj.2025.10.002.

REFERENCES

1. Iskratsch, T., H. Wolfenson, and M. P. Sheetz. 2014. Appreciating force and shape—the rise of mechanotransduction in cell biology. *Nat. Rev. Mol. Cell Biol.* 15:825–833.
2. Sackmann, E., and A.-S. Smith. 2014. Physics of cell adhesion: some lessons from cell-mimetic systems. *Soft Matter.* 10:1644–1659.
3. Engler, A. J., S. Sen, …, D. E. Discher. 2006. Matrix elasticity directs stem cell lineage specification. *Cell.* 126:677–689.
4. Wen, J. H., L. G. Vincent, …, A. J. Engler. 2014. Interplay of matrix stiffness and protein tethering in stem cell differentiation. *Nat. Mater.* 13:979–987.
5. Dupont, S., L. Morsut, …, S. Piccolo. 2011. Role of YAP/TAZ in mechanotransduction. *Nature.* 474:179–183.
6. Elosegui-Artola, A., I. Andreu, …, P. Roca-Cusachs. 2017. Force triggers YAP nuclear entry by regulating transport across nuclear pores. *Cell.* 171:1397–1410.e14.
7. Hino, N., L. Rossetti, …, T. Hirashima. 2020. ERK-mediated mechanochemical waves direct collective cell polarization. *Dev. Cell.* 53:646–660.e8.
8. Hundsdorfer, L., M. Muenkel, …, E. E. Bastounis. 2025. ERK activation waves coordinate mechanical cell competition leading to collective elimination via extrusion of bacterially infected cells. *Cell Rep.* 44:115193.

9. Boocock, D., N. Hino, …, E. Hannezo. 2021. Theory of mechanochemical patterning and optimal migration in cell monolayers. *Nat. Phys.* 17:267–274.
10. Boocock, D., T. Hirashima, and E. Hannezo. 2023. Interplay between mechanochemical patterning and glassy dynamics in cellular monolayers. *PRX Life.* 1:013001.
11. Yu, P., R. Zhang, and B. Li. 2025. Cell Stiffness-Mediated Mechanochemical Waves in Three-Dimensional Tissues. *Phys. Rev. Lett.* 135:108401.
12. Das, T., K. Safferling, …, J. P. Spatz. 2015. A molecular mechanotransduction pathway regulates collective migration of epithelial cells. *Nat. Cell Biol.* 17:276–287.
13. Heisenberg, C.-P., and Y. Bellaïche. 2013. Forces in tissue morphogenesis and patterning. *Cell.* 153:948–962.
14. Hannezo, E., and C.-P. Heisenberg. 2019. Mechanochemical feedback loops in development and disease. *Cell.* 178:12–25.
15. Brodland, G. W., V. Conte, …, M. Miodownik. 2010. Video force microscopy reveals the mechanics of ventral furrow invagination in Drosophila. *Proc. Natl. Acad. Sci. USA.* 107:22111–22116.
16. Allena, R., J. J. Muñoz, and D. Aubry. 2013. Diffusion-reaction model for Drosophila embryo development. *Comput. Methods Biomech. Biomed. Eng.* 16:235–248.
17. Mercker, M., A. Köthe, and A. Marciniak-Czochra. 2015. Mechanochemical symmetry breaking in Hydra aggregates. *Biophys. J.* 108:2396–2407.
18. Maroudas-Sacks, Y., S. Suganthan, …, K. Keren. 2025. Mechanical strain focusing at topological defect sites in regenerating Hydra. *Development (Camb.).* 152:DEV204514.
19. Ravichandran, Y., M. Vogg, …, A. Roux. 2025. Topology changes of Hydra define actin orientation defects as organizers of morphogenesis. *Sci. Adv.* 11:eadr9855.
20. Bailles, A., G. Serafini, …, P. Tomancak. 2025. Anisotropic stretch biases the self-organization of actin fibers in multicellular Hydra aggregates. *Proc. Natl. Acad. Sci. USA.* 122:e2423437122.
21. Blanchoin, L., R. Boujemaa-Paterski, …, J. Plastino. 2014. Actin dynamics, architecture, and mechanics in cell motility. *Physiol. Rev.* 94:235–263.
22. Sackmann, E. 2015. How actin/myosin crosstalks guide the adhesion, locomotion and polarization of cells. *Biochim. Biophys. Acta.* 1853:3132–3142.
23. Banerjee, S., M. L. Gardel, and U. S. Schwarz. 2020. The actin cytoskeleton as an active adaptive material. *Annu. Rev. Condens. Matter Phys.* 11:421–439.
24. Prost, J., F. Jülicher, and J.-F. Joanny. 2015. Active gel physics. *Nat. Phys.* 11:111–117.
25. Jülicher, F., K. Kruse, …, J.-F. Joanny. 2007. Active behavior of the cytoskeleton. *Phys. Rep.* 449:3–28.
26. Recho, P., T. Putelat, and L. Truskinovsky. 2013. Contraction-Driven Cell Motility. *Phys. Rev. Lett.* 111:108102.
27. Drozdowski, O. M., F. Ziebert, and U. S. Schwarz. 2023. Optogenetic control of migration of contractile cells predicted by an active gel model. *Commun. Phys.* 6:158.
28. Ishihara, S., P. Marcq, and K. Sugimura. 2017. From cells to tissue: A continuum model of epithelial mechanics. *Phys. Rev.* 96:022418.
29. Beta, C., L. Edelstein-Keshet, …, A. Yochelis. 2023. From actin waves to mechanism and back: How theory aids biological understanding. *eLife.* 12:e87181.
30. Riveline, D., E. Zamir, …, A. D. Bershadsky. 2001. Focal contacts as mechanosensors: externally applied local mechanical force induces growth of focal contacts by an mDia1-dependent and ROCK-independent mechanism. *J. Cell Biol.* 153:1175–1186.
31. Bement, W. M., M. Leda, …, G. von Dassow. 2015. Activator–inhibitor coupling between Rho signalling and actin assembly makes the cell cortex an excitable medium. *Nat. Cell Biol.* 17:1471–1483.
32. Bischof, J., C. A. Brand, …, P. Lénárt. 2017. A cdk1 gradient guides surface contraction waves in oocytes. *Nat. Commun.* 8:849.
33. Liu, J., T. Burkart, …, N. Fakhri. 2025. Light-induced cortical excitability reveals programmable shape dynamics in starfish oocytes. *Nat. Phys.* 21:846–855.
34. Kamps, D., J. Koch, …, L. Dehmelt. 2020. Optogenetic Tuning Reveals Rho Amplification-Dependent Dynamics of a Cell Contraction Signal Network. *Cell Rep.* 33:108467.
35. Katsumi, A., J. Milanini, …, M. A. Schwartz. 2002. Effects of cell tension on the small GTPase Rac. *J. Cell Biol.* 158:153–164.
36. He, L., J. Tao, …, S. X. Sun. 2018. Role of membrane-tension gated Ca2+ flux in cell mechanosensation. *J. Cell Sci.* 131:jcs208470.
37. Houk, A. R., A. Jilkine, …, O. D. Weiner. 2012. Membrane tension maintains cell polarity by confining signals to the leading edge during neutrophil migration. *Cell.* 148:175–188.
38. Besser, A., and U. S. Schwarz. 2007. Coupling biochemistry and mechanics in cell adhesion: A model for inhomogeneous stress fiber contraction. *New J. Phys.* 9:425.
39. Zmurchok, C., D. Bhaskar, and L. Edelstein-Keshet. 2018. Coupling mechanical tension and GTPase signaling to generate cell and tissue dynamics. *Phys. Biol.* 15:046004.
40. Buttenschön, A., Y. Liu, and L. Edelstein-Keshet. 2020. Cell size, mechanical tension, and GTPase signaling in the single cell. *Bull. Math. Biol.* 82:1–33.
41. Staddon, M. F., E. M. Munro, and S. Banerjee. 2022. Pulsatile contractions and pattern formation in excitable actomyosin cortex. *PLoS Comput. Biol.* 18:e1009981.
42. Weitzman, M., and K. M. Hahn. 2014. Optogenetic approaches to cell migration and beyond. *Curr. Opin. Cell Biol.* 30:112–120.
43. Boyden, E. S., F. Zhang, …, K. Deisseroth. 2005. Millisecond-timescale, genetically targeted optical control of neural activity. *Nat. Neurosci.* 8:1263–1268.
44. Konermann, S., M. D. Brigham, …, F. Zhang. 2013. Optical control of mammalian endogenous transcription and epigenetic states. *Nature.* 500:472–476.
45. Wang, X., X. Chen, and Y. Yang. 2012. Spatiotemporal control of gene expression by a light-switchable transgene system. *Nat. Methods.* 9:266–269.
46. Zhao, E. M., Y. Zhang, …, J. L. Avalos. 2018. Optogenetic regulation of engineered cellular metabolism for microbial chemical production. *Nature.* 555:683–687.
47. Valon, L., F. Etoc, …, M. Coppey. 2015. Predictive Spatiotemporal Manipulation of Signaling Perturbations Using Optogenetics. *Biophys. J.* 109:1785–1797.
48. Valon, L., A. Marín-Llauradó, …, X. Trepat. 2017. Optogenetic control of cellular forces and mechanotransduction. *Nat. Commun.* 8:14396.
49. Oakes, P. W., E. Wagner, …, M. L. Gardel. 2017. Optogenetic control of RhoA reveals zyxin-mediated elasticity of stress fibres. *Nat. Commun.* 8:15817.
50. Andersen, T., D. Wörthmüller, …, M. Balland. 2023. Cell size and actin architecture determine force generation in optogenetically activated cells. *Biophys. J.* 122:684–696.
51. Hinderling, L., A. E. Landolt, …, O. Pertz. 2025. Real-time feedback control microscopy for automation of optogenetic targeting. Preprint at bioRxiv. https://doi.org/10.1101/2025.08.17.670729.
52. Ruppel, A., D. Wörthmüller, …, M. Balland. 2023. Force propagation between epithelial cells depends on active coupling and mechanostructural polarization. *eLife.* 12:e83588.
53. Rossetti, L., S. Grosser, …, X. Trepat. 2024. Optogenetic generation of leader cells reveals a force–velocity relation for collective cell migration. *Nat. Phys.* 20:1659–1669.
54. Marée, A. F. M., A. Jilkine, …, L. Edelstein-Keshet. 2006. Polarization and movement of keratocytes: a multiscale modelling approach. *Bull. Math. Biol.* 68:1169–1211.

55. Edwards, C. M., and U. S. Schwarz. 2011. Force localization in contracting cell layers. *Phys. Rev. Lett.* 107:128101.

56. Mertz, A. F., S. Banerjee, …, E. R. Dufresne. 2012. Scaling of traction forces with the size of cohesive cell colonies. *Phys. Rev. Lett.* 108:198101.

57. Banerjee, S., and M. Cristina Marchetti. 2013. Controlling cell-matrix traction forces by extracellular geometry. *New J. Phys.* 15:035015.

58. Solowiej-Wedderburn, J., and C. M. Dunlop. 2022. Sticking around: Cell adhesion patterning for energy minimization and substrate mechanosensing. *Biophys. J.* 121:1777–1786.

59. Trepat, X., M. R. Wasserman, …, J. J. Fredberg. 2009. Physical forces during collective cell migration. *Nat. Phys.* 5:426–430.

60. Tambe, D. T., U. Croutelle, …, J. J. Fredberg. 2013. Monolayer stress microscopy: limitations, artifacts, and accuracy of recovered intercellular stresses. *PLoS One.* 8:e55172.

61. Bauer, A., M. Prechová, …, B. Fabry. 2021. pyTFM: A tool for traction force and monolayer stress microscopy. *PLoS Comput. Biol.* 17:e1008364.

62. Chojowski, R., U. S. Schwarz, and F. Ziebert. 2024. The role of the nucleus for cell mechanics: an elastic phase field approach. *Soft Matter.* 20:4488–4503.

63. Nishikawa, M., S. R. Naganathan, …, S. W. Grill. 2017. Controlling contractile instabilities in the actomyosin cortex. *eLife.* 6:e19595.

64. Mercker, M., F. Brinkmann, …, T. Richter. 2016. Beyond Turing: Mechanochemical pattern formation in biological tissues. *Biol. Direct.* 11:22.

65. Hippler, M., K. Weißenbruch, …, M. Bastmeyer. 2020. Mechanical stimulation of single cells by reversible host-guest interactions in 3D microscaffolds. *Sci. Adv.* 6:eabc2648.

66. Ern, A., A. F. Stephansen, and P. Zunino. 2008. A discontinuous Galerkin method with weighted averages for advection–diffusion equations with locally small and anisotropic diffusivity. *IMA J. Numer. Anal.* 29:235–256.

67. Beguerisse-Díaz, M., R. Desikan, and M. Barahona. 2016. Linear models of activation cascades: Analytical solutions and coarse-graining of delayed signal transduction. *J. R. Soc. Interface.* 13:20160409.

68. Saha, A., M. Nishikawa, …, S. W. Grill. 2016. Determining physical properties of the cell cortex. *Biophys. J.* 110:1421–1429.

69. Crellin, H. A., C. Zhu, …, C. E. Buckley. 2024. Local optogenetic NMYII activation within the zebrafish neural rod results in long-range, asymmetric force propagation. Preprint at bioRxiv. https://doi.org/10.1101/2024.09.19.613826v4.

70. Guglielmi, G., J. D. Barry, …, S. De Renzis. 2015. An Optogenetic Method to Modulate Cell Contractility during Tissue Morphogenesis. *Dev. Cell.* 35:646–660.

71. Izquierdo, E., T. Quinkler, and S. De Renzis. 2018. Guided morphogenesis through optogenetic activation of Rho signalling during early Drosophila embryogenesis. *Nat. Commun.* 9:2366.

Biophysical Journal, Volume *125*

Supplemental information

Modeling mechanochemical coupling in optogenetically activated cell layers

Dennis Wörthmüller, Falko Ziebert, and Ulrich S. Schwarz

Modelling mechanochemical coupling in optogenetically activated cell layers

- Supplemental text -

D. Wörthmüller, F. Ziebert and U.S. Schwarz

General notation

In solid mechanics one typically expresses model equations in the Lagrangian (material) framework by describing a deforming solid in terms of coordinates $\hat{\mathbf{x}}$ representing the position of material particles in the undeformed configuration Ω_0. The current position of the material particles in the deformed configuration $\Omega(t)$ at time t are given by $\mathbf{x} = \boldsymbol{\chi}(\hat{\mathbf{x}}, t)$. The two configurations are connected by the displacement vector field $\hat{\mathbf{u}}(\hat{\mathbf{x}}, t) = \mathbf{x}(\hat{\mathbf{x}}, t) - \hat{\mathbf{x}}$. The deformation gradient tensor is defined as $\hat{\mathbf{F}} = \partial\mathbf{x}/\partial\hat{\mathbf{x}} = \mathbf{I} + \hat{\nabla}\hat{\mathbf{u}}$ and measures the local change of relative position of two points at the transition from the undeformed to the deformed configuration. Its determinant $\hat{J} = \det(\hat{\mathbf{F}})$ represents the local volume change. Describing the governing equations in terms of the coordinates $\mathbf{x}$ is known as the Eulerian framework where a fixed point in space is observed. In the limit of linear elasticity we do not distinguish between Lagrangian and Eulerian description and drop the $\wedge$-symbol.

Reaction-diffusion on time dependent domains in Lagrangian frame of reference

For the pull-back of

$$\frac{\partial c_i}{\partial t} + \nabla \cdot (\mathbf{v}(\mathbf{x}, t)c_i) = \nabla \cdot (\mathbf{D}\nabla c_i)) + R_{c_i}(t) \tag{S1}$$

to the reference configuration (Lagrangian coordinates) we exploit the transformation rules for scalar and vector fields as well as the involved differential operators (1). For mappings between the two reference systems we use the motion function $\mathbf{x} = \boldsymbol{\chi}(\hat{\mathbf{x}}, t)$. Each Eulerian field has a Lagrangian counterpart which states the equivalence of the two descriptions. Hence we write $c(\mathbf{x}, t) = \hat{c}(\hat{\mathbf{x}}, t)$ for the concentration field and $\mathbf{v}(\mathbf{x}, t) = \hat{\mathbf{v}}(\hat{\mathbf{x}}, t)$ for the velocity field. Here $\hat{\mathbf{v}} = \partial_t\boldsymbol{\chi}(\hat{x}, t)$ denotes the material velocity. For the gradient of the scalar field $c(\mathbf{x}, t)$ it holds

$$\nabla c = \hat{\mathbf{F}}^{-\intercal}\hat{\nabla}\hat{c} \, . \tag{S2}$$

For the gradient of the vector field $\mathbf{v}$ we have

$$\nabla\mathbf{v} = \hat{\nabla}\hat{\mathbf{v}}\hat{\mathbf{F}}^{-1} \; . \tag{S3}$$

The divergence of the vector field transforms according to

$$\nabla\cdot\mathbf{v} = \frac{1}{\hat{J}}\hat{\nabla}\cdot(\hat{J}\hat{\mathbf{F}}^{-1}\hat{\mathbf{v}}) \; , \tag{S4}$$

and we note that Piola's identity is given by

$$\hat{\nabla}\cdot(\hat{J}\hat{\mathbf{F}}^{-1}) = 0 \; . \tag{S5}$$

The material time derivative of a field expressed in Eulerian coordinates is given by the convective derivative

$$\frac{\mathrm{d}}{\mathrm{d}t}c(\mathbf{x},t) = \frac{\partial}{\partial t}c + \mathbf{v}\cdot\nabla c \equiv \frac{\partial\hat{c}}{\partial t} \tag{S6}$$

Starting from the reaction-diffusion equation in Eulerian coordinates

$$\frac{\partial c_i}{\partial t} + \mathbf{v}\cdot\nabla c_i + c_i(\nabla\cdot\mathbf{v}) = \nabla\cdot(\mathbf{D}\nabla c_i)) + R_{c_i}(t) \; , \tag{S7}$$

we note that the first two terms correspond to the convective time derivative. The third term on the left and the first term on the right hand side of the equation can be replaced by using the Piola transform Eq. (S4) such that

$$\frac{\partial\hat{c}_i}{\partial t} + \hat{c}_i\frac{1}{\hat{J}}\hat{\nabla}\cdot(\hat{J}\hat{\mathbf{F}}^{-1}\hat{\mathbf{v}}) = \frac{1}{\hat{J}}\hat{\nabla}\cdot(\hat{J}\hat{\mathbf{F}}^{-1}\mathbf{D}\hat{\mathbf{F}}^{-\intercal}\hat{\nabla}\hat{c}_i)) + R_{\hat{c}_i}(t) \; , \tag{S8}$$

and after multiplying by $\hat{J}$ we obtain

$$\hat{J}\frac{\partial\hat{c}_i}{\partial t} + \hat{c}_i\hat{\nabla}\cdot(\hat{J}\hat{\mathbf{F}}^{-1}\hat{\mathbf{v}}) = \hat{\nabla}\cdot(\hat{J}\hat{\mathbf{F}}^{-1}\mathbf{D}\hat{\mathbf{F}}^{-\intercal}\hat{\nabla}\hat{c}_i)) + \hat{J}R_{\hat{c}_i}(t) \; . \tag{S9}$$

Applying Piola's identity to the second term on the left we find

$$\hat{c}_i\hat{\nabla}\cdot(\hat{J}\hat{\mathbf{F}}^{-1}\hat{\mathbf{v}}) = \hat{c}_i\hat{J}\hat{\mathbf{F}}^{-1}\hat{\nabla}\hat{\mathbf{v}} = \hat{c}_i\hat{J}\hat{\mathbf{F}}^{-1}\frac{\partial\hat{\mathbf{F}}}{\partial t} = \hat{c}_i\frac{\partial\det(\hat{\mathbf{F}})}{\partial t} = \hat{c}_i\frac{\partial\hat{J}}{\partial t} \; , \tag{S10}$$

and further using $\hat{\mathbf{C}} = \hat{\mathbf{F}}^{\intercal}\hat{\mathbf{F}}$ we end up with

$$\frac{\partial}{\partial t}(\hat{J}\hat{c}_i) = \hat{\nabla}\cdot(\hat{J}\mathbf{D}\hat{\mathbf{C}}^{-1}\hat{\nabla}\hat{c}_i) + \hat{J}R_{\hat{c}_i}(t) \; . \tag{S11}$$

Finite-Element formulation

Symmetric Weighted Interior Penalty Discontinuous Galerkin method

The interface condition on the cell-cell junction

$$-\hat{J}\mathbf{D}\hat{\mathbf{C}}^{-1}\hat{\nabla}\hat{c}_i\cdot\mathbf{N}_{\mathsf{CCJ}} = 0 \quad \text{on} \;\; \Gamma_{\mathsf{CCJ}} \; , \tag{S12}$$

leads to a discontinuity in the solution. In order to account for abrupt concentration changes from one subdomain to the other we need a method which allows discontinuous functions across the membrane. The standard choice for such a problem is the discontinuous Galerkin method. In contrast to the continuous Galerkin methods, continuity and smoothness of the involved DG-functions is only enforced element-wise such that the solution may be discontinuous across element boundaries. If and where desired, continuity can be enforced through an appropriate penalty term. These methods are known as interior penalty discontinuous Galerkin methods (IPDG) (2–4). However, as can be seen from Eq. (S11) the diffusivity $\boldsymbol{\alpha} \equiv \hat{J}\mathbf{D}\hat{\mathbf{C}}^{-1}$ in the Lagrangian frame is position dependent due to local deformations of the domain. Hence, we are dealing with heterogeneous diffusion and use a Symmetric Weighted Interior Penalty (SWIP) discontinuous Galerkin scheme (5). Let $\mathcal{T}(\Omega_0)$ be the triangulation of the domain Ω into finite elements $e \in \mathcal{T}(\Omega_0)$. Further, let $\mathcal{F}$ denote the union of the boundary facets of all elements e. We distinguish between external facets $\mathcal{F}_{\text{ext}}$, internal facets $\mathcal{F}_{\text{int}}$ and membrane facets $\mathcal{F}_{\text{M}}$ such that $\mathcal{F} = \mathcal{F}_{\text{ext}} \cup \mathcal{F}_{\text{int}} \cup \mathcal{F}_{\text{M}}$ with $\mathcal{F}_{\text{int}} = \mathcal{F} \setminus (\mathcal{F}_{\text{ext}} \cup \mathcal{F}_{\text{M}})$. Next, by $\hat{c}_-$ and $\hat{c}_+$ we denote scalar valued functions on two neighboring elements e_- and e_+. The normal vectors on a common facet of $e_\pm$ are given by $\mathbf{N}_\pm$. For example, $\mathbf{N}_-$ defines the outward directed normal on e_- pointing into e_+. Following the SWIP-DG notations we introduce the jump and the weighted average of a quantity as $[\![\hat{c}]\!] \equiv \hat{c}_+\mathbf{N}_+ + \hat{c}_-\mathbf{N}_-$ and $\{\hat{c}\}_\omega \equiv \omega_+\hat{c}_+ + \omega_-\hat{c}_-$, respectively. Analogously, for piecewise vector valued functions $\hat{\mathbf{q}}$ one defines jump and weighted average as $[\![\hat{\mathbf{q}}]\!] \equiv \hat{\mathbf{q}}_+\mathbf{N}_+ + \hat{\mathbf{q}}_-\mathbf{N}_-$ and $\{\hat{\mathbf{q}}\}_\omega \equiv \omega_+\hat{\mathbf{q}}_+ + \omega_-\hat{\mathbf{q}}_-$, respectively. The weights are defined as $\omega_\pm = \delta_\alpha^\mp/(\delta_\alpha^+ + \delta_\alpha^-)$, where $\delta_\alpha^\mp$ is obtained from the diffusivity on two neighbouring elements $e_\mp$ by calculating $\delta_\alpha^\mp = \tilde{\mathbf{N}}_e^\mathsf{T}\boldsymbol{\alpha}_\mp\tilde{\mathbf{N}}_e$. Note that the weights fulfill $\omega_+ + \omega_- = 1$. Since we are only interested in having a discontinuity across the membrane facets $\mathcal{F}_{\text{M}}$ we introduce a diffusion dependent penalty term to penalize jumps across all other internal facets $\mathcal{F}_{\text{int}}$ which is defined as the harmonic mean $\gamma_\alpha = 2\delta_\alpha^+\delta_\alpha^-/(\delta_\alpha^+ + \delta_\alpha^-)$. Moreover, one may use these definitions to prove the identity

$$[\![\hat{\mathbf{q}}\hat{c}]\!] = [\![\hat{\mathbf{q}}]\!]\{\hat{c}\}_\omega + \{\hat{\mathbf{q}}\}_\omega[\![\hat{c}]\!] \; . \tag{S13}$$

In the first step of the derivation of the DG weak form we multiply Eq. (S11) with a suitable test function $v_c \in \mathcal{V}$ and integrate over the whole simulation domain Ω_0 which gives

$$\begin{aligned} \int_{\Omega_0} \frac{\partial}{\partial t}(\hat{J}\hat{c}) v_c \; \mathrm{d}\Omega_0 - \int_{\Omega_0} \hat{\nabla} \cdot \left(\boldsymbol{\alpha}\hat{\nabla}\hat{c}\right) v_c \; \mathrm{d}\Omega_0 & \\ - \int_{\Omega_0} \hat{J} R_c(t) v_c \; \mathrm{d}\Omega_0 = 0 \; . & \end{aligned} \tag{S14}$$

Instead of directly using partial integration on the middle term of Eq. (S14) we first split it into a sum over element integrals and then apply Green's first theorem to obtain

$$\begin{aligned} \int_{\Omega_0} \hat{\nabla} \cdot \left(\boldsymbol{\alpha}\hat{\nabla}\hat{c}\right) v_c \; \mathrm{d}\Omega_0 &= \sum_{e \in \mathcal{T}(\Omega_0)} \int_e \hat{\nabla} \cdot \left(\boldsymbol{\alpha}\hat{\nabla}\hat{c}\right) v_c \; \mathrm{d}\Omega_0 \\ &= \sum_{f_e \in \mathcal{F}(\Omega_0)} \int_{f_e} \boldsymbol{\alpha}\hat{\nabla}\hat{c} \cdot \tilde{\mathbf{N}}_e v_c \; \mathrm{d}s \\ &\quad - \sum_{e \in \mathcal{T}(\Omega_0)} \int_e \boldsymbol{\alpha}\hat{\nabla}\hat{c} \cdot \hat{\nabla} v_c \; \mathrm{d}\Omega_0 \; . \end{aligned} \tag{S15}$$

Here, f_e denotes the facets of element e and $\tilde{\mathbf{N}}_e$ describes the outward directed normal vector on the facets of the element. The first term in Eq. (S15) is split again into the exterior, interior and membrane facets

$$\sum_{f_e \in \mathcal{F}(\Omega_0)} \int_{f_e} \boldsymbol{\alpha} \hat{\nabla} \hat{c} \cdot \tilde{\mathbf{N}}_e v_c \, \mathrm{d}s = \sum_{f_e \in \mathcal{F}_{\text{ext}}(\Omega_0)} \int_{f_e} \boldsymbol{\alpha} \hat{\nabla} \hat{c} \cdot \tilde{\mathbf{N}}_e v_c \, \mathrm{d}s + \sum_{f_e \in \mathcal{F}_{\text{int}}(\Omega_0)} \int_{f_e} \boldsymbol{\alpha} \hat{\nabla} \hat{c} \cdot \tilde{\mathbf{N}}_e v_c \, \mathrm{d}s + \sum_{f_e \in \mathcal{F}_{\text{M}}(\Omega_0)} \int_{f_e} \boldsymbol{\alpha} \hat{\nabla} \hat{c} \cdot \tilde{\mathbf{N}}_e v_c \, \mathrm{d}s \, . \tag{S16}$$

Note that each internal facet and each membrane facet is shared by two adjacent elements e_- and e_+ (see Fig. S1) such that integrals along the common facets add up to a jump

$$\int_{f_\pm} \boldsymbol{\alpha} \hat{\nabla} \hat{c} \cdot \tilde{\mathbf{N}}_\pm v_c \, \mathrm{d}s = \int_f (\delta_\alpha^+ \hat{\nabla} \hat{c}_+ v_{c,+} - \delta_\alpha^- \hat{\nabla} \hat{c}_- v_{c,-}) \cdot \tilde{\mathbf{N}}_+ \, \mathrm{d}s = \int_f [\![\boldsymbol{\alpha} \hat{\nabla} \hat{c} v_c]\!] \, \mathrm{d}s \, . \tag{S17}$$

Summing up over all elements e in Eq. (S15) and Eq. (S16) while respecting zero-flux boundary conditions yields

$$\int_{\Omega_0} \hat{\nabla} \cdot \left(\boldsymbol{\alpha} \hat{\nabla} \hat{c} \right) v_c \, \mathrm{d}\Omega_0 = - \int_{\Omega_0} \boldsymbol{\alpha} \hat{\nabla} \hat{c} \cdot \hat{\nabla} v_c \, \mathrm{d}\Omega_0 + \int_{\mathcal{F}_{\text{int}}} [\![\boldsymbol{\alpha} \hat{\nabla} \hat{c} v_c]\!] \, \mathrm{d}s \, . \tag{S18}$$

The last term in Eq. (S18) is further expanded using the identity in Eq. (S13) which yields

$$\int_{\mathcal{F}_{\text{int}}} [\![\boldsymbol{\alpha} \hat{\nabla} \hat{c} v_c]\!] \, \mathrm{d}s = \int_{\mathcal{F}_{\text{int}}} [\![\boldsymbol{\alpha} \hat{\nabla} \hat{c}]\!] \cdot \{ v_c \}_\omega \, \mathrm{d}s + \int_{\mathcal{F}_{\text{int}}} \{ \boldsymbol{\alpha} \hat{\nabla} \hat{c} \}_\omega \cdot [\![v_c]\!] \, \mathrm{d}s \, . \tag{S19}$$

Since the exact solution of the diffusion equation is expected to be smooth we enforce continuity of the fluxes by setting $[\![\boldsymbol{\alpha} \hat{\nabla} \hat{c}]\!] = 0$. To further enforce continuity of the solution we exploit $[\![\hat{c}]\!] = 0$ and add a term to symmetrize the problem. Additionally, we ensure stability of the problem by adding a stabilizing term according to Ern et al. (5) and Douglas and Dupont (6) which finally leads to

$$\int_{\mathcal{F}_{\text{int}}} [\![\boldsymbol{\alpha} \hat{\nabla} \hat{c} v_c]\!] \, \mathrm{d}s = \int_{\mathcal{F}_{\text{int}}} \{ \boldsymbol{\alpha} \hat{\nabla} \hat{c} \}_\omega \cdot [\![v_c]\!] \, \mathrm{d}s + \int_{\mathcal{F}_{\text{int}}} \{ \boldsymbol{\alpha} \hat{\nabla} v_c \}_\omega \cdot [\![\hat{c}]\!] \, \mathrm{d}s - \int_{\mathcal{F}_{\text{int}}} \frac{s_N}{h} \gamma_\alpha [\![\hat{c}]\!] \cdot [\![v_c]\!] \, \mathrm{d}s \, . \tag{S20}$$

In Eq. (S20), s_N denotes the so-called Nitsche paramater, which must be chosen sufficiently large to ensure continuity across internal facets (7), and h the average element diameter. Next, we define

$$\mathcal{D}(\hat{c}, v_c, \boldsymbol{\alpha}) := \int_{\Omega_0} \boldsymbol{\alpha} \hat{\nabla} \hat{c} \cdot \hat{\nabla} v_c \, \mathrm{d}\Omega_0 - \int_{\mathcal{F}_{\text{int}}} \{ \boldsymbol{\alpha} \hat{\nabla} \hat{c} \}_\omega \cdot [\![v_c]\!] \, \mathrm{d}s - \int_{\mathcal{F}_{\text{int}}} \{ \boldsymbol{\alpha} \hat{\nabla} v_c \}_\omega \cdot [\![\hat{c}]\!] \, \mathrm{d}s + \int_{\mathcal{F}_{\text{int}}} \frac{s_N}{h} \gamma_\alpha [\![\hat{c}]\!] \cdot [\![v_c]\!] \, \mathrm{d}s \, , \tag{S21}$$

and hence arrive at the final weak form statement of Eq. (S11) which reads

$$\int_{\Omega_0} \frac{\partial}{\partial t} (\hat{J} \hat{c}) v_c \, \mathrm{d}\Omega_0 + \mathcal{D}(\hat{c}, v_c, \boldsymbol{\alpha}) - \int_{\Omega_0} \hat{J} R_c(t) v_c \, \mathrm{d}\Omega_0 = 0 \, . \tag{S22}$$

We note that an integral part of our model design is that continuity is only enforced on the internal edges. Thus jumps are possible only across the interface of the two subdomains i.e. across the membrane. In our case this means that the reactants in each cell cannot pass the cell-cell junction. This leads to a variety of possibilities in the treatment of multicellular systems. Cellular contractility in principle can be described by distinct reaction-diffusion systems in each cell. The RD-systems within the cells can then be coupled by appropriate mechano-chemical coupling terms to account for mechanosensing at the intercellular junction.

The weak form of Eq. (S11) finally reads

$$
\begin{aligned}
0 = &\int_{\Omega_0} \frac{\partial}{\partial t}(\hat{J}\hat{c}) v_c \, \mathrm{d}\Omega_0 + \int_{\Omega_0} \boldsymbol{\alpha} \hat{\nabla} \hat{c} \cdot \hat{\nabla} v_c \, \mathrm{d}\Omega_0 \\
&- \underbrace{\int_{\mathcal{F}_{\text{int}}} \{\boldsymbol{\alpha} \hat{\nabla} \hat{c}\}_\omega \cdot [\![v_c]\!] \, \mathrm{d}s}_{\text{consistency}} - \underbrace{\int_{\mathcal{F}_{\text{int}}} \{\boldsymbol{\alpha} \hat{\nabla} v_c\}_\omega \cdot [\![\hat{c}]\!] \, \mathrm{d}s}_{\text{symmetry}} + \underbrace{\int_{\mathcal{F}_{\text{int}}} \frac{s_N}{h} \gamma_\alpha [\![\hat{c}]\!] \cdot [\![v_c]\!] \, \mathrm{d}s}_{\text{penalty}} - \int_{\Omega_0} \hat{J} R_c(t) v_c \, \mathrm{d}\Omega_0 \, ,
\end{aligned}
$$

where s_N denotes the Nitsche paramater, which must be chosen sufficiently large to ensure continuity across internal facets (7), and h the average element diameter. The notation is illustrated in Fig. S1a.

Weak formulation for the elastic domain

To derive the weak formulation of

$$\nabla \cdot \boldsymbol{\sigma} = Y(\mathbf{x})\mathbf{u} \, , \tag{S23}$$

we multiply with a vector valued test function $\mathbf{v} \in \boldsymbol{\mathcal{V}}(\Omega_0)$ and integrate over the domain Ω_0 of the undeformed configuration

$$\int_{\Omega_0} (\nabla \cdot \boldsymbol{\sigma}) \cdot \mathbf{v} \, \mathrm{d}\Omega_0 = \int_{\Omega_0} Y(\mathbf{x})\mathbf{u}(\mathbf{x}) \cdot \mathbf{v} \, \mathrm{d}\Omega_0 \, . \tag{S24}$$

The left hand side can be integrated using integration by parts i.e. using the following identity

$$\nabla \cdot (\boldsymbol{\sigma} \cdot \mathbf{v}) = (\nabla \cdot \boldsymbol{\sigma}) \cdot \mathbf{v} + \boldsymbol{\sigma} : \nabla \mathbf{v} \, . \tag{S25}$$

This allows to simplify Eq. (S24) to

$$\int_{\Omega_0} \boldsymbol{\sigma} : \nabla \mathbf{v} \, \mathrm{d}\Omega_0 - \int_{\Gamma} (\boldsymbol{\sigma} \cdot \mathbf{N}) \cdot \mathbf{v} \, \mathrm{d}s + \int_{\Omega_0} Y \mathbf{u} \cdot \mathbf{v} \, \mathrm{d}\Omega_0 = 0 \, . \tag{S26}$$

Here, $\boldsymbol{\sigma} \cdot \mathbf{N}$ is the traction vector at the boundary $\Gamma = \partial\Omega_0$ which is set to zero in case of stress free boundaries and hence, the final weak form statement reads

$$\int_{\Omega_0} \boldsymbol{\sigma} : \nabla \mathbf{v} \, \mathrm{d}\Omega_0 + \int_{\Omega_0} Y \mathbf{u} \cdot \mathbf{v} \, \mathrm{d}\Omega_0 = 0 \, . \tag{S27}$$

Time-discretisation

All time dependent quantities $Q(t)$ are discretized using an implicit (backward Euler) scheme at a given time $t^{(n+1)}$

$$\left(\frac{\mathrm{d}Q}{\mathrm{d}t}\right)^{(n+1)} \approx \frac{Q^{(n+1)} - Q^{(n)}}{\Delta t} \,. \tag{S28}$$

Mesh generation

The meshes for the three different systems were generated with Gmsh (9). For the cell chain and the tissue-like monolayer we made sure to create a mesh which respects the symmetry of the system. The meshes are shown in Fig. S1c.

Literature review RhoA-pathway

Having reviewed the relevant literature, we found that the model as presented by Kamps et al. (8) contains all important components which are necessary for a profound description of the RhoA pathway. In contrast to the RhoA-actomyosin system as introduced by Staddon et al. (10) it explicitly contains GEF as a downstream effector of RhoA, and thus provides an important interface for light-induced contraction as GEF activity can be controlled by optogenetic constructs like the CRY2/CIBN system. In combination with experimental measurements Kamps et al. (8) proposed a reaction scheme for the active reactants GEF (G), RhoA (R) and myosin (M) which reads

$$\frac{\mathrm{d}G}{\mathrm{d}t} = k_3 R(G_T - G) - k_4 G M \tag{S29}$$

$$\frac{\mathrm{d}R}{\mathrm{d}t} = \frac{k_1 G(R_T - R)}{K_{m1} + R_T - R} - k_2 \frac{R}{K_{m2} + R} \tag{S30}$$

$$\frac{\mathrm{d}M}{\mathrm{d}t} = \frac{k_5 R(M_T - M)}{K_{m5} + M_T - M} - k_6 \frac{M}{K_{m6} + M} \,. \tag{S31}$$

G_T, R_T and M_T denote the total concentrations of the species which the authors assume to be constant. The rate constants are denoted by k_i and the Michaelis-Menten constants are given by the K_{mi}.

The membrane and cytosol associated species represent the active and passive states, respectively. This terminology stems from experimental studies which show that the active forms of RhoA and myosin are predominantly found in the vicinity of the plasma membrane and the submembraneous actin cortex. In contrast, the inactive forms are associated with the cytosol (11, 12). The RhoA protein for example exhibits a lipophilic end which enables it to bind to lipid membranes (13). However, so-called guanosine dissociation inhibitors (GDIs) may bind to Rho-GDPs, not only keeping them in a permanently inactive state but also preventing its membrane localization by shielding the hydrophilic end and additionally making it soluble in the cytoplasm (14). The reaction scheme also highlights the two feedback loops which are important to describe the excitable and oscillatory dynamics that are

observed in experiments (see Fig. S1 d). The positive feedback loop stems from the observation that RhoA activity at the membrane further induces GEF membrane recruitment. Due to Rho activation by GEFs, this closes a positive feedback loop (8). The negative feedback loop can be traced back to the ability of myosin to inhibit the nucleotide exchange activity of GEFs by binding to their Dbl-homology domain (DH) (15). Essentially, the authors could identify the total concentration of active GEF as the main bifurcation parameter for the switch from stable to oscillatory states at intermediate GEF concentrations. In experiments, they vary this bifurcation parameter by treating cells with nocodazole, which leads to depolymerization of microtubules from which GEFs are then released. The crossover from stable to osciallatory dynamics happens as a function of the total GEF concentration.

Parametrization

Non-linear RhoA pathway

For the simulations with the non-linear Rho pathway we follow Kamps et al. (8). In Eqs. (S29) to (S31) we treat the inactive species separately with $c_i = c_T - c$. The inactive species diffuse faster than the active species $D_{c,i} > D_c$. The reaction kinetics are given by $R_{c_i} = -R_c$. Hence, for the non-linear system we have a total of six coupled reaction diffusion equations coupled to the PDE describing the cell layer. Fig. S1d shows the schematic of the coupled system of PDE's. The reaction diffusion system alone exhibits instabilities that lead to the emergence of traveling wave peaks. To induce an instability we impose initial conditions on the active species by adding small random fluctuations of the form

$$c_0(\mathbf{x}) = \bar{c}_0 + \delta c_0(0.5 - \mathcal{U}_{[0,1](\mathbf{x})}) \,, \tag{S32}$$

where $\mathcal{U}_{[0,1](\mathbf{x})}$ is the probability density function of the continuous uniform distribution, $\bar{c}_0$ the homogeneous concentration field and δc_0 a small flucutation. For the inactive species we set $c_{i,0}(\mathbf{x}) = c_T - c_0(\mathbf{x})$. Fluctuations are $\delta G_0 = 0.05$, $\delta R_0 = 0.01$ and $\delta M_0 = 0$. All other relevant parameters can be found in Tab. S1 or in the supplemental information of (8).

Linearized RhoA pathway

For the parametrization of the proposed linear signaling cascade we rely on the order of magnitudes found in the respective literature. Within the limits of our simplified model the total concentrations of RhoA and myosin are irrelevant since they do not explicitly enter the reaction kinetics in the weakly activated regime. The parameters are chosen such that the steady state concentrations of RhoA and myosin are roughly $10\,\%$ of the total concentration (10, 16). Further, the reaction rates are chosen such that the time course of the myosin concentration approximates the typical time course of actively generated stresses during optogenetic activation (8, 10, 17–19). The time course of the input signal was adapted to the measured CRY2 membrane recruitment and is described by a relaxation time of $\approx 10^2\,\mathrm{s}$ ($\lambda \approx 0.01\,\mathrm{s}^{-1}$)(20). The reference values from (8) were estimated as follows. The second term in Eq. (S30) can be approximated in a weakly activated regime with $K_{m2} \gg R$ as $k_2 R/(K_{m2} + R) \approx k_2 R/K_{m2} \equiv b \approx 2\,\mathrm{s}^{-1}$. The same argument applied to the second term in

Eq. (S31) gives $k_6 M/(K_{m6}+M) \approx k_6 M/K_{m6} \equiv s \approx 0.0051\,\mathrm{s}^{-1}$. k can be estimated from the first term in Eq. (S31) by $k_5/(K_{m5}+M_T) = \tilde{k} = 0.004\,65\,\mathrm{s}^{-1}$ from which $k = \tilde{k} R_T = 0.002\,\mathrm{s}^{-1}$ follows. All relevant parameters are summarized in Tab. S2.

Elastic layer

For the parametrisation of the cell and the substrate we follow the typical orders of magnitude. Cell and substrate have a Young's modulus E in the range of several kPa. For simplicity we choose $E_c \approx E_s$, which also reflects that cells typically adapt to the stiffness of their environment. The viscoelastic time scale τ_c is a free parameter in the simulations and hence the viscosity of the cell layer is defined by $\eta_c = \tau_c E_c$. The spring stiffness density is calculated by $Y_s = \pi E_s / L_c$ (21), where L_c is the lateral extent of a cell. All other relevant parameters are summarized in Tables S3 to S5.

Analytical solution of the weakly activated signaling cascade

Beguerisse-Díaz et al. (22) provide a variety of analytical solutions to weakly activated signalling cascades triggered by different input signals i.e. time course of the stimulus such as step-function, Gaussian or, as in our case, an exponential decreasing perturbation. The signaling species x_1^* is activated by an external stimulus, which in turn activates species x_2^*, and so on. In a weakly activated regime and for $x_i^*(0) = 0$ (which is the initial condition for the perturbations δr and δm) the output function of species x_n^* is given by

$$x_n^*(t) = \left(\prod_{i=1}^{n} \alpha_i\right) \sum_{i=1}^{n} \left(\prod_{q=1, q\neq i}^{n} (\beta_i - \beta_q)^{-1}\right) \times \int_0^t e^{-\beta_i (t-\tau)} A(\tau)\ \mathrm{d}\tau \ . \tag{S33}$$

Here α_i and β_i denote the activation and deactivation rates of each species i, respectively, and $A(t)$ is a stimulus applied to the first species. Applied to the system of equations

$$\frac{\mathrm{d}\delta r}{\mathrm{d}t} = a\delta g(t) - b\delta r \ , \quad \frac{\mathrm{d}\delta m}{\mathrm{d}t} = k\delta r - s\delta m \ , \tag{S34}$$

together with $A(\tau) = \delta g(\tau) = g_a e^{-\lambda\tau}$ (as in the main text) this leads to

$$\delta\tilde{r}(t) = \frac{\delta r}{r_{\mathsf{ss}}} = \frac{b\alpha}{b-\lambda}\left(e^{-\lambda t} - e^{-bt}\right) \ , \tag{S35}$$

$$\delta\tilde{m}(t) = \frac{\delta m}{m_{\mathsf{ss}}} = b\alpha s\left(\frac{e^{-\lambda t} - e^{-bt}}{(b-s)(b-\lambda)} - \frac{e^{-\lambda t} - e^{-st}}{(b-s)(s-\lambda)}\right) \ . \tag{S36}$$

Description of movies

Movie S1: Strain-dependent feedback in a cell doublet with strong coupling. Activation of the left cell leads to substantial contraction in the right cell. Although optogenetic stimulation is only applied to the left cell, the whole doublet contracts as a unit i.e. symmetrically. Parameters used as listed in Tab. S2 and Tab. S3 with $a_{\delta\tilde{q}} = 100$ and $\tau_c = 10\text{s}$.

Movie S2: Strain-dependent feedback in a cell doublet with weak coupling. Activation of the left cell leads to weak contraction in the right cell. This leads to an overall asymmetric shape deformation of the whole doublet in which the right cell gets pulled to the left. Parameters used as listed in Tab. S2 and Tab. S3 with $a_{\delta\tilde{q}} = 0.1$ and $\tau_c = 10\text{s}$.

Movie S3: Propagation of a contraction wave through a cell chain visualized by the deformation field (color code: red color corresponds to displacement to the right, blue to the left). Optogenetic activation of the left cell leads to a contraction. The coupling to the neighboring cell induces a contraction which propagates from the left end of the cell chain to the right end. Parameters used as listed in Tab. S2 and Tab. S4. Coupling and viscoelastic time scale where chosen from the transmissive regime. Red lines indicate initial cell-cell boundary positions.

Movie S4: Propagation of contraction wave through a tissue-like monolayer comprised of 28 cells. Here, the contractility is controlled by the non-linear Rho-pathway which in itself exhibits wave-like instabilities. The parameters are chosen according to Tab. S1 and Tab. S5. At early times, an instability is triggered in the centered cell on the left by adding small random fluctuations on the GEF and Rho component. These random fluctuations lead to several concentrated traveling wave peaks which in turn trigger GEF activation in adjacent cells through the strain-dependent coupling. Thus, a contraction wave spreads through the whole tissue activating all cells and triggering the wave-like instability. The emerging activation pattern represents the up-down symmetry of the tissue. Cells at the free edge (edge with no cell-cell boundary) strongly deform when a contraction peak gets close to the free cell edge.

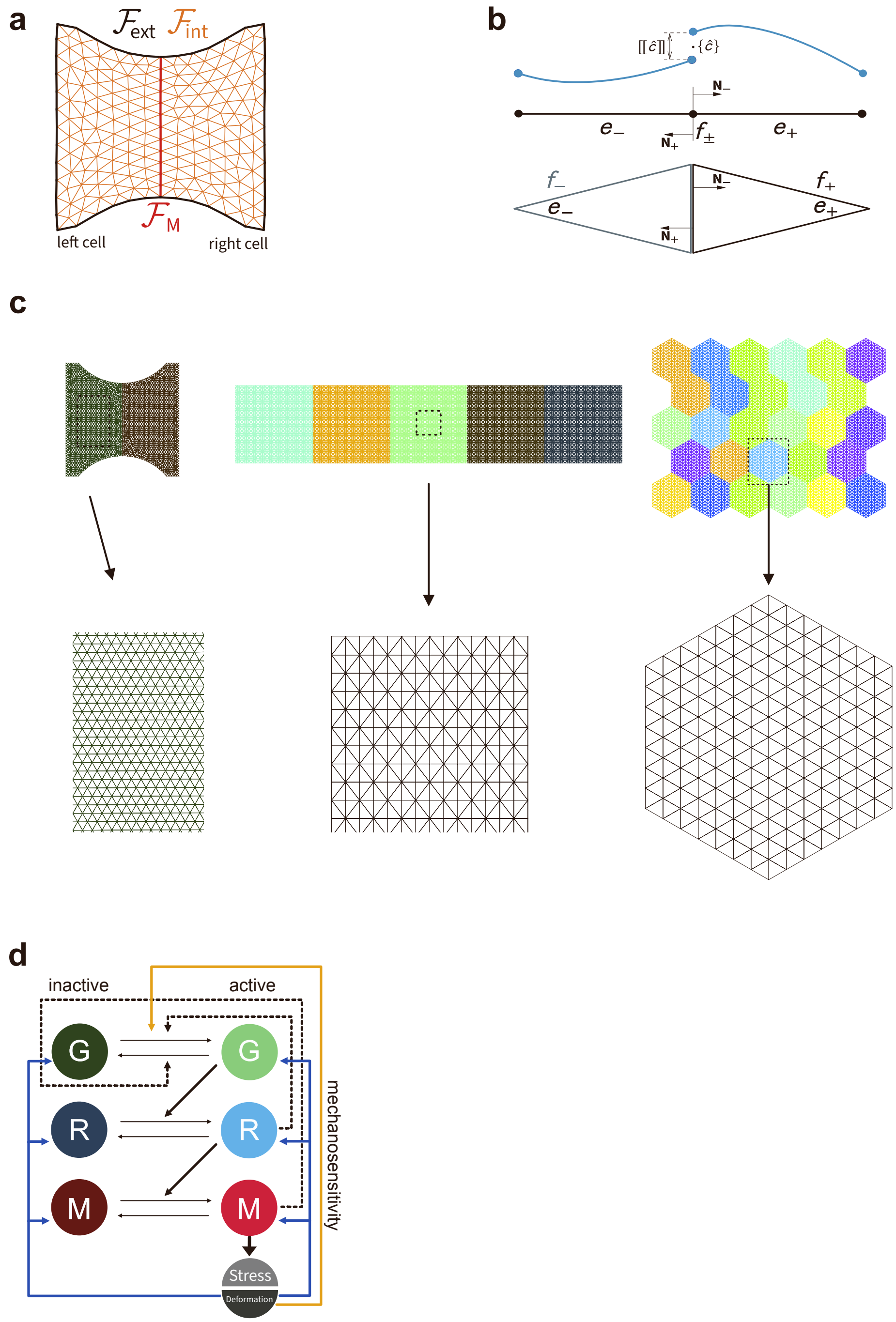

Figure S1: Panel (a) and (b) schematically explains the notation used in the derivation of the discontinuous Galerkin finite element method. Panel (c) depicts the meshes for the three cell systems. Colors highlight different cells. For cell chain and tissue-like monolayer we enforced meshes to respect the symmetry of the system's geometry. Panel (d) shows the coupled system of the non-linear RhoA pathway by Kamps et al. (8) coupled to the mechanics of the elastic layer. Mechanical feedbacks represented by yellow and blue arrows.

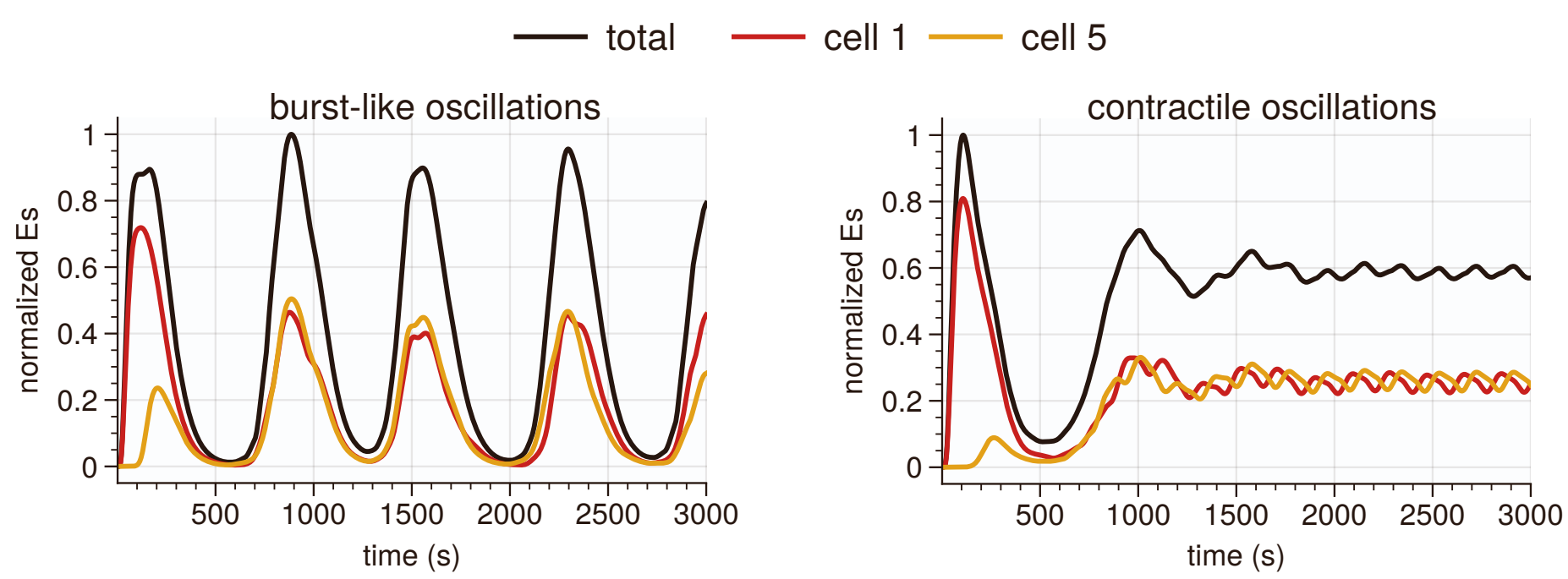

Figure S2: Different oscillations observed in a cell chain coupled to the linearized RhoA pathway. Burst-like oscillations (left) correspond to large variations in strain energy while contractile oscillations (right) corresponds to states in which the whole cell chain remains on average in a state of non-vanishing strain energy.

Abbreviation	Value
	GEF
G_T	$0.2\mathrm{M}$
k_3	$1.19\mathrm{M}^{-1}\mathrm{s}^{-1}$
k_4	$3.98\mathrm{M}^{-1}\mathrm{s}^{-1}$
D_G	$0.3\,\mu\mathrm{m}^2\,\mathrm{s}^{-1}$
D_{G_i}	$9.28\,\mu\mathrm{m}^2\,\mathrm{s}^{-1}$
	RhoA
R_T	$0.443\mathrm{M}$
K_{m1}	$2.42\mathrm{M}$
K_{m2}	$0.0745\mathrm{M}$
k_1	$(3.88K_{m1})\mathrm{s}^{-1}$
k_2	$(2.04K_{m2})\mathrm{Ms}^{-1}$
D_R	$0.3\,\mu\mathrm{m}^2\,\mathrm{s}^{-1}$
D_{R_i}	$9.28\,\mu\mathrm{m}^2\,\mathrm{s}^{-1}$
	Myosin
M_T	$1.24\mathrm{M}$
K_{m5}	$0.014\mathrm{M}$
K_{m6}	$0.784\mathrm{M}$
k_5	$(0.417K_{m5})\mathrm{s}^{-1}$
k_6	$(0.00509K_{m9})\mathrm{Ms}^{-1}$
D_M	$0.03\,\mu\mathrm{m}^2\,\mathrm{s}^{-1}$
D_{M_i}	$0.9\,\mu\mathrm{m}^2\,\mathrm{s}^{-1}$

Table S1: Parameter values for the non-linear Rho pathway with fast and slow diffusing species according to (8). Here, we use M to indicate units of concentration which corresponds to 10^6 molecules per cell (as given in the SI of (8)).

Abbreviation	Used value	Ref. value	Reference
λ	$0.01\,\mathrm{s}^{-1}$	$0.008\,\mathrm{s}^{-1} - 0.018\,\mathrm{s}^{-1}$	(20)
α	$100\,\%$	$20\,\% - 130\,\%$	(20)
b	$0.0165\,\mathrm{s}^{-1}$	$2\,\mathrm{s}^{-1}$	(8)
k	$0.1\,\mathrm{s}^{-1}$	$0.1408\,\mathrm{s}^{-1}$	(10)
		$0.002\,\mathrm{s}^{-1}$	(8)
s	$0.083\,\mathrm{s}^{-1}$	$0.0051\,\mathrm{s}^{-1}$	(8)
		$0.082\,\mathrm{s}^{-1}$	(10)
m_{ss}	0.1	$0.1 - 0.8$	(10)
			(16)
D_R, D_G	$0.3\,\mu\mathrm{m}^2\,\mathrm{s}^{-1}$	$0.28\,\mu\mathrm{m}^2\,\mathrm{s}^{-1}$	(8)
		$0.1\,\mu\mathrm{m}^2\,\mathrm{s}^{-1}$	(23)
D_M	$0.03\,\mu\mathrm{m}^2\,\mathrm{s}^{-1}$	$0.03\,\mu\mathrm{m}^2\,\mathrm{s}^{-1}$	(8)
		$0.01\,\mu\mathrm{m}^2\,\mathrm{s}^{-1}$	(23)
Deduced			
r_{ss}	0.083		
a	$0.0014\,\mathrm{s}^{-1}$	$< 0.002\,\mathrm{s}^{-1}$	(10)

Table S2: Parameter values for the linearized RhoA signaling cascade. We set most of the parameters in accordance with the reported ranges. The parameters taken from (8) where obtained by taking the corresponding activation and deactivation rates in a weakly activated regime for which the Michaelis-Menten terms can be linearly approximated. However, their model does not provide a basal activation rate. The reported rate constants as stated in the work of Staddon et al. (10) were deduced from Michaux et al. (24). The parameters r_{ss} and a are not independent and consequently deduced from the fixed parameters.

Abbreviation	Value
**Cell parameters**	
Young's Modulus E_c	$5 \cdot 10^3 \mathrm{Pa}$
Poisson's ratio ν_c	0.5
Cell height h_c	$1 \cdot 10^{-6} \mathrm{m}$
Lateral extent L_c	$45 \cdot 10^{-6} \mathrm{m}$
Two-dimensional active stress σ_0	$2 \cdot 10^{-3} \mathrm{N\,m}^{-1}$
**Substrate parameters**	
Young's Modulus E_s	$5 \cdot 10^3 \mathrm{Pa}$
Poisson's ratio ν_s	0.5

Table S3: Parameter values for the simulation of the cell doublet on an H-pattern

Abbreviation	Value
**Cell parameters**	
Young's Modulus E_c	$5 \cdot 10^3 \mathrm{Pa}$
Poisson's ratio ν_c	0.5
Cell height h_c	$1 \cdot 10^{-6} \mathrm{m}$
Lateral extent L_c	$40 \cdot 10^{-6} \mathrm{m}$
Two-dimensional active stress σ_0	$2 \cdot 10^{-3} \mathrm{N\,m}^{-1}$
**Substrate parameters**	
Young's Modulus E_s	$2.5 \cdot 10^3 \mathrm{Pa}$
Poisson's ratio ν_s	0.5

Table S4: Parameter values for the simulation of the cell chain with continuous adhesion and unidirectional contraction.

Abbreviation	Value
Cell parameters	
Young's Modulus E_c	$2.5 \cdot 10^3 \mathrm{Pa}$
Poisson's ratio ν_c	0.5
Cell height h_c	$1 \cdot 10^{-6} \mathrm{m}$
Lateral extent L_c	$40 \cdot 10^{-6} \mathrm{m}$
Two-dimensional active stress σ_0	$2.5 \cdot 10^{-3} \mathrm{N\,m^{-1}}$
Substrate parameters	
Young's Modulus E_s	$1 \cdot 10^3 \mathrm{Pa}$
Poisson's ratio ν_s	0.5

Table S5: Parameter values for the simulation of the tissue-like monolayer with non-linear Rho-pathway. Here, we chose a smaller Young's modulus for the cell, a softer substrate and a slightly larger value for active stress in order to allow for more visible deformations.

Example for python FEM-code for photoactivation of a cell doublet on H-pattern

Our model was implemented in the FEM-framework FEniCS (25). This is an example code for the photoactivation of a cell doublet with mechanochemical feedback.

```python
# Import required libraries for finite element analysis and data processing
from dolfin import *          # FEniCS/DOLFIN for finite element computations
import numpy as np            # Numerical computations
import random                 # Random number generation
import copy                   # Deep copying of objects
import matplotlib.pyplot as plt  # Plotting utilities
from ufl import tanh         # Hyperbolic tangent function
from numpy import savetxt    # Array saving utility
import pandas as pd          # Data manipulation and analysis
import os                    # Operating system interface
import csv                   # CSV file operations

''' Finite-Element-Simulation of a coupled system of reaction-diffusion equations and 2D
    viscoelasticity

    This code implements a coupled mechanochemical model for cell mechanics using FEM.
    The model combines:
    1. Reaction-diffusion equations for biochemical species
    2. 2D viscoelastic mechanics for cell deformation
    3. Mechanochemical feedback between strain and signaling

    Copyright: Dennis Woerthmueller
    Date: February 14, 2024
'''

# Create data directory for simulation outputs
path_to_rawData = 'Data/'
if not os.path.exists(path_to_rawData):
    os.makedirs(path_to_rawData)

# Load simulation parameters from CSV file
with open('inputParams.csv', newline = '') as file:
    reader = csv.reader(file, quoting = csv.QUOTE_NONNUMERIC,
                        delimiter = ' ')
    rows = list(reader)
    keys = rows[0]     # Parameter names
    values = rows[1]   # Parameter values

# Create parameter dictionary from keys and values
key_value_pairs = zip(keys, values)
p = dict(key_value_pairs)

def calculateStrainEnergy(u, kN, dx):
    """
    Calculate the elastic strain energy in the substrate.

    Args:
        u (Function): Displacement field
        kN (Expression): Spring constant field
        dx (Measure): Integration measure

    Returns:
        float: Total strain energy
    """
    return assemble(0.5*kN*inner(u,u)*dx)

class KNExpression(UserExpression):
```

```
    """
    Define position-dependent spring constants for an H-shaped micropattern.

    The pattern consists of two vertical arms connected by a horizontal crossbar.
    Spring constants are non-zero only within the pattern.
    """
    def __init__(self,Y, armWidth, degree=2):
        print("BIS HIER")
        super().__init__()
        self.armWidth = armWidth  # Width of pattern arms
        self.Y = Y               # Young’s modulus / spring constant

    def eval(self, value, x):
        """
        Evaluate spring constant at given position.

        Args:
            value: Output value (modified in-place)
            x: Spatial coordinates
        """
        d = 1  # Domain size
        # Set spring constant Y in arms and crossbar, 0 elsewhere
        if (x[0] <= -(d/2)+self.armWidth or x[0] >= (d/2)-self.armWidth or
            between(x[1],(-self.armWidth/2,self.armWidth/2))):
            value[0] = self.Y
        else:
            value[0] = 0.0

def normalize_solution(U, max):
    """
    Normalize solution vector by dividing by maximum value.

    Args:
        U (Function): Solution vector to normalize
        max (float): Maximum value to normalize by

    Returns:
        Function: Normalized solution vector
    """
    U_array = U.vector().get_local()
    U_array /= max
    U.vector()[:] = U_array
    return U

def eps(v):
    """
    Calculate strain tensor from displacement field.

    Args:
        v (Function): Displacement field

    Returns:
        Tensor: Symmetric gradient (strain tensor)
    """
    return sym(grad(v))

class selectedSubdomain(UserExpression):
    """
    Mark specific subdomains with different values.
    Used to identify and assign properties to different regions.
    """
    def __init__(self, subdomains, val_inside, val_outside, subdomain_id, **kwargs):
        super().__init__()
        self.subdomains = subdomains        # Subdomain markers
        self.val_inside = val_inside        # Value inside selected subdomain
        self.val_outside = val_outside      # Value outside selected subdomain
```

```
        self.subdomain_id = subdomain_id    # ID of subdomain to mark

    def eval_cell(self, values, x, cell):
        """
        Evaluate marker value for each cell.

        Args:
            values: Output value (modified in-place)
            x: Spatial coordinates
            cell: Current cell
        """
        if self.subdomains[cell.index] == self.subdomain_id:
            values[0] = self.val_inside
        else:
            values[0] = self.val_outside

class SquareCompartmentDoublet(UserExpression):
    """
    Define square compartment for cell doublet simulation.
    Used to create initial conditions and activation patterns.
    """
    def __init__(self,A,d, degree=0):
        super().__init__()
        self.A = A  # Amplitude
        self.d = d  # Distance/size parameter

    def eval(self, value, x):
        """
        Evaluate compartment value at given position.

        Args:
            value: Output value (modified in-place)
            x: Spatial coordinates
        """
        if (x[0] <= -self.d):
            value[0] = self.A
        else:
            value[0] = 0.0

def get_boundary_of_deformed_mesh(u, geo_file_name):
    """
    Extract boundary coordinates of deformed mesh.
    Used for tracking boundary deformation over time.

    Args:
        u (Function): Displacement field
        geo_file_name (str): Base name of geometry files

    Returns:
        numpy.array: Sorted boundary coordinates
    """
    # Load mesh and boundary definitions
    dummy_mesh = Mesh("%s.xml"%(geo_file_name))
    boundaries = MeshFunction("size_t", dummy_mesh, "%s_facet_region.xml"%(geo_file_name))
    subdomains = MeshFunction("size_t", dummy_mesh, "%s_physical_region.xml"%(geo_file_name))

    # Apply displacement to mesh
    ALE.move(dummy_mesh, u)
    V_mesh = FunctionSpace(dummy_mesh, "CG", 1)
    v2d = vertex_to_dof_map(V_mesh)

    # Extract boundary vertices
    dofs = []
    for facet in facets(dummy_mesh):
        if boundaries[facet.index()] == 2:  # Top boundary
            vertices = facet.entities(0)
```

```python
            for vertex in vertices:
                dofs.append(v2d[vertex])

    # Sort and return boundary coordinates
    unique_dofs = np.array(list(set(dofs)), dtype=np.int32)
    boundary_coords = V_mesh.tabulate_dof_coordinates()[unique_dofs]
    col = 0
    boundary_coords_sorted = boundary_coords[np.argsort(boundary_coords[:,col])]
    return boundary_coords_sorted

def DGWeakFormRD(c, cn, vc, Dc, u, J, J_n, F, n, dx, dS, dSM, dt, cellularisation=True):
    """
    Construct weak form for reaction-diffusion equations using Discontinuous Galerkin method.

    Args:
        c (Function): Current concentration
        cn (Function): Previous concentration
        vc (TestFunction): Test function
        Dc (float): Diffusion coefficient
        u (Function): Displacement field
        J (Expression): Current Jacobian
        J_n (Expression): Previous Jacobian
        F (Expression): Deformation gradient
        n (Expression): Normal vector
        dx (Measure): Volume measure
        dS (Measure): Interior facet measure
        dSM (Measure): Interface measure
        dt (float): Time step
        cellularisation (bool): Whether to include cell interface terms

    Returns:
        Form: Complete weak form for reaction-diffusion equation
    """
    # Calculate geometric quantities
    F_inv = inv(F)
    F_inv_T = inv(F).T
    I = Identity(2)
    eps = sym(grad(u))

    # Numerical parameters
    h = 0.1   # Mesh size parameter
    sN = 50   # Penalty parameter

    # Modified diffusion tensor including geometric factors
    alph = J*(I-2*eps)*Dc

    # Calculate interface terms for DG formulation
    delt_p = dot(n('+'), alph('+')*n('+'))
    delt_m = dot(n('-'), alph('-')*n('-'))

    # Weights for averaging
    w_pos = delt_m/(delt_p+delt_m)
    w_neg = delt_p/(delt_p+delt_m)

    # Average terms for concentration gradients
    grad_c_avg_term = w_pos*alph('+')*grad(c)('+')+w_neg*alph('-')*grad(c)('-')
    grad_vc_avg_term = w_pos*alph('+')*grad(vc)('+')+w_neg*alph('-')*grad(vc)('-')

    # Interface penalty parameter
    gamma = 2*delt_p*delt_m/(delt_p+delt_m)

    # Time derivative terms
    time_deriv = J*(c-cn)/dt*vc*dx+(J-J_n)/dt*c*vc*dx

    # Standard diffusion term
    standard = dot(alph * grad(c), grad(vc)) * dx
```

```python

    # Interface terms based on cellularisation flag
    if cellularisation:
        # Terms for internal cell boundaries
        consistency = -dot(jump(vc,n),grad_c_avg_term)*dS + dot(jump(vc,n),grad_c_avg_term)*dSM
        symmetry = - dot(grad_vc_avg_term, jump(c,n))*dS + dot(grad_vc_avg_term,jump(c,n))*dSM
        penalty = sN/h*gamma*dot(jump(vc, n), jump(c,n))* dS - sN/h*gamma*dot(jump(vc,n), jump(c, n))*dSM
    else:
        # Terms without internal boundaries
        consistency = -dot(jump(vc,n),grad_c_avg_term)*dS
        symmetry = - dot(grad_vc_avg_term, jump(c,n))*dS
        penalty = sN/h*gamma*dot(jump(vc, n), jump(c,n))* dS

    # Combine all terms
    weakForm = time_deriv + standard + consistency + symmetry + penalty

    return weakForm

# Output file names
name = 'simulation_output'           # Name of the output xdmf-file
geo_file_name = 'cell_doublet_shape_nonDim'  # Name of gmsh .geo-file

def simulation():
    """
    Main simulation function implementing a mechanochemical feedback model.

    The simulation couples three main components:
    1. Reaction-diffusion system for GEF-RhoA-Myosin signaling
    2. Viscoelastic mechanics for cell deformation
    3. Mechanochemical feedback through strain

    Uses mixed finite elements: DG for concentrations, CG for displacement.
    """
    # --------------------------------------------------------------------------
    # Initialize mesh and finite element structures
    # --------------------------------------------------------------------------
    # Convert mesh from Gmsh format to FEniCS XML format
    if not os.path.exists('%s.xml'%(geo_file_name)):
        os.system('dolfin-convert %s.msh %s.xml'%(geo_file_name,geo_file_name))

    # Load mesh and domain definitions
    mesh = Mesh("%s.xml"%(geo_file_name))
    boundaries = MeshFunction("size_t", mesh, "%s_facet_region.xml"%(geo_file_name))
    subdomains = MeshFunction("size_t", mesh, "%s_physical_region.xml"%(geo_file_name))

    # Save domain definitions for visualization
    file_results = XDMFFile("subdomains.xdmf")
    file_results.write(subdomains)
    file_results = XDMFFile("boundaries.xdmf")
    file_results.write(boundaries)

    # Define measures for integration
    dx = Measure('dx', domain=mesh, subdomain_data=subdomains)    # Volume measure
    dS_all = Measure('dS', subdomain_data=boundaries)            # Surface measure
    dSM = dS_all(1)                                             # No-flux interface measure

    # Initialize domain markers
    cell1 = selectedSubdomain(subdomains, 1, 0, subdomain_id = 1, degree=0)       # Active cell
    non_opto_cell = selectedSubdomain(subdomains, 0, 1, subdomain_id = 1, degree=0) # Non-photoactivated cell
    n = FacetNormal(mesh)                                                         # Normal vector field

    # Define finite elements for mixed formulation
    P1 = FiniteElement('DG', triangle, 1)           # DG elements for concentrations
```

```
    P2 = VectorElement('CG', triangle, 1)           # CG elements for displacement
    element = MixedElement([P1, P1, P1,P2])         # Combined element
    V = FunctionSpace(mesh, element)                 # Mixed function space

    # Initialize function spaces for output fields
    # DG spaces for scalar fields
    dFE_DG0 = FiniteElement("DG", mesh.ufl_cell(), 0)
    dFE_DG1 = FiniteElement("DG", mesh.ufl_cell(), 1)
    dFE_CG1 = FiniteElement("CG", mesh.ufl_cell(), 1)
    dFE_CG2 = FiniteElement("CG", mesh.ufl_cell(), 2)

    # Tensor spaces for stress/strain fields
    TensorSpace_DG0 = TensorFunctionSpace(mesh, "DG", 0)
    TensorSpace_DG1 = TensorFunctionSpace(mesh, "DG", 1)
    TensorSpace_CG1 = TensorFunctionSpace(mesh, "CG", 1)
    TensorSpace_CG2 = TensorFunctionSpace(mesh, "CG", 2)

    # Create function spaces
    W_DG0 = TensorSpace_DG0     # DG0 tensor space
    W_DG1 = TensorSpace_DG1     # DG1 tensor space
    W_CG1 = TensorSpace_CG1     # CG1 tensor space
    W_CG2 = TensorSpace_CG2     # CG2 tensor space

    # Scalar function spaces
    K_DG0 = FunctionSpace(mesh, dFE_DG0)
    K_DG1 = FunctionSpace(mesh, dFE_DG1)
    K_CG1 = FunctionSpace(mesh, dFE_CG1)
    K_CG2 = FunctionSpace(mesh, dFE_CG2)

    # Vector function space for displacement
    V_CG1 = VectorFunctionSpace(mesh,"CG",1)

    # Initialize output functions
    # Vector fields (displacement and traction)
    disp = Function(V_CG1, name='Displacement')
    TractionF = Function(V_CG1, name='Traction')

    # Scalar fields for molecular species
    GEF = Function(K_DG0, name='GEF')                     # GEF concentration
    RhoA = Function(K_DG0, name='RhoA')                   # RhoA concentration
    Myosin = Function(K_DG0, name='Myosin')             # Myosin concentration
    GEF_inactive = Function(K_DG0, name='GEF inactive')
    RhoA_inactive = Function(K_DG0, name='RhoA inactive')
    Myosin_inactive = Function(K_DG0, name='Myosin inactive')

    # Scalar fields for mechanics
    activatedCell = Function(K_DG0, name='Activated Cell')
    nonOptoCells = Function(K_DG0, name='Non-opto Cells')
    pattern = Function(K_DG0, name='Micropattern')
    Jacobian = Function(K_DG0, name='detF')
    JacobianPositive = Function(K_DG0, name='detF +')
    feedbackPositive = Function(K_DG0, name='feedback')
    hypTangentPositive = Function(K_DG0, name='tanh')
    traceGreenLagrange = Function(K_DG0, name='trE')
    traceGreenLagrangePositive = Function(K_DG0, name='trE +')
    detCauchyStressPositive = Function(K_DG0, name='det(CS) +')

    # Tensor fields for stress and strain
    Cauchystress = Function(W_DG0, name='Cauchy Stress')
    Cauchystress_passive = Function(W_DG0, name='Passive Cauchy Stress')
    Pstress = Function(W_DG0, name='Piola1 Stress')
    Pstress_passive = Function(W_DG0, name='Passive Piola1 Stress')
    activeStress = Function(W_DG0, name='Active Stress')
    strainGreenLagrange = Function(W_DG0, name='E (GL Strain)')
    defGrad_save = Function(W_DG0, name='Deformation Gradient Tensor')
    CauchyGreenInverse_save = Function(W_DG0, name='Inverse Cauchy Green')
```

```
    # Diffusion tensors
    diffTensor_G = Function(W_DG0, name='alpha_G')
    diffTensor_R = Function(W_DG0, name='alpha_R')
    diffTensor_M = Function(W_DG0, name='alpha_M')

    # Initialize output file
    xdmf_file = XDMFFile(path_to_rawData+"%s.xdmf"%(name))
    xdmf_file.parameters["flush_output"] = True
    xdmf_file.parameters["functions_share_mesh"] = True

    # Define spring constant field for substrate
    kN = KNExpression(p['Ys_N'], p['armWidth_N'], degree=2)

    # Set time discretization parameters
    DT = 0.5                  # Time step size
    dt = Constant(DT)         # FEniCS constant for time step
    t = DT                    # Current time
    T = 500                   # End time
    t_opto = 5                 # photoactivation

    # Initialize variational problem components
    dU = TrialFunction(V)     # Trial function
    U_tot = Function(V)       # Current solution
    U_tot_n = Function(V)     # Previous solution
    vG, vR, vM, vu = TestFunctions(V)  # Test functions

    # Additional functions for solution storage
    U_tot_save = Function(V)
    u_save = Function(V_CG1)
    U_tot_save_n = Function(V)

    # Set initial conditions
    u0 = Constant((0.0,0.0))      # Zero displacement
    random.seed()                  # Set random seed
    G_0 = Constant(0)             # Initial GEF concentration
    R_0 = Constant(0)             # Initial RhoA concentration
    M_0 = Constant(0)             # Initial Myosin concentration

    # Project initial conditions to appropriate function spaces
    uG_n = project(G_0, V.sub(0).collapse())
    uR_n = project(R_0, V.sub(1).collapse())
    uM_n = project(M_0, V.sub(2).collapse())
    u_n = project(u0, V.sub(3).collapse())

    # Assign initial conditions to solution vector
    assign(U_tot_n, [uG_n,uR_n,uM_n,u_n])

    # Split solution for component access
    uG,uR, uM, u = split(U_tot)           # Current solution
    uG_n, uR_n, uM_n, u_n = split(U_tot_n)  # Previous solution

    # Initialize arrays for storing results
    time_array = []
    strainEnergyLeft_vs_time = []
    strainEnergyRight_vs_time = []
    strainEnergyTotal_vs_time = []
    boundary_curve_vs_time = []



    # Main time stepping loop
    while t <= T:
        print("TIME = ", t)

        # ---------------------------------------------------------------------------------------
```

```
        # Set up tensors for continuum description of the cell layer
        # -------------------------------------------------------------------------------------
        # Define fundamental geometric tensors
        I = Identity(2)                     # Identity tensor
        F = I + grad(u)                     # Deformation gradient tensor
        J_n = 1+tr(grad(u_n))              # Previous Jacobian (volume change)
        J = 1+tr(grad(u))                  # Current Jacobian
        F_transpose = F.T                   # Transpose of deformation gradient
        F_inv = I - grad(u)                 # Approximate inverse of deformation gradient
        C = I - 2*sym(grad(u))             # Right Cauchy-Green tensor
        C1 = inv(C)                         # Inverse of Cauchy-Green tensor

        # Calculate strain measures
        eps_n = sym(grad(u_n))             # Previous strain tensor
        eps = sym(grad(u))                 # Current strain tensor
        trace_n = tr(eps_n)                # Previous volumetric strain
        trace = tr(eps)                    # Current volumetric strain

        # -------------------------------------------------------------------------------------
        # Define active stresses from myosin activity
        # -------------------------------------------------------------------------------------
        # Calculate myosin-dependent active stress
        activeStress_from_myosin = p['sig_a_N']* tanh(1*uM)  # Nonlinear myosin activation
        # Convert to tensor form (isotropic active stress)
        activeStress_tensor = activeStress_from_myosin*as_tensor([[1, 0], [0, 1]])

        # -------------------------------------------------------------------------------------
        # Constitutive relations for viscoelastic material
        # -------------------------------------------------------------------------------------
        # Total Cauchy stress including active and passive components
        CS = (activeStress_tensor +                                    # Active stress
              p['lmbdaE_N'] * tr(eps) * Identity(2) +                 # Elastic volumetric
              2*p['muE_N']*eps +                                      # Elastic deviatoric
              p['tauc']*p['lmbdaE_N']*(trace-trace_n)/dt*Identity(2) + # Viscous volumetric
              2*p['muE_N']*p['tauc']*(eps-eps_n)/dt)                  # Viscous deviatoric

        # Passive component of Cauchy stress
        CS_passive = (p['lmbdaE_N'] * tr(eps) * Identity(2) +         # Elastic volumetric
                     2*p['muE_N']*eps +                               # Elastic deviatoric
                     p['tauc']*p['lmbdaE_N']*(trace-trace_n)/dt*Identity(2) + # Viscous volumetric
                     2*p['muE_N']*p['tauc']*(eps-eps_n)/dt)           # Viscous deviatoric

        # Calculate positive and negative parts for mechanochemical feedback
        trace_positive = conditional(gt(trace,0),trace,0)*non_opto_cell    # Positive strain
        trace_negative = conditional(gt(0,trace),trace,0)*non_opto_cell    # Negative strain
        J_positive = conditional(gt(J-1,0),J-1,0)*non_opto_cell           # Positive volume change
        detCS_positive = conditional(gt(0,det(CS_passive)),det(CS_passive),0)   # Positive stress determinant

        # Calculate traction force
        tF = kN*u                             # Linear spring force
        tF_mag = kN*sqrt(inner(u,u))         # Magnitude of traction force
        tF_mag_projected = project(tF_mag, K_DG0)  # Project for visualization

        # -------------------------------------------------------------------------------------
        # Handle photoactivation event
        # -------------------------------------------------------------------------------------
        if t == t_opto:
            # Initialize photoactivation pattern
            G_0 = Constant(p['alpha'])*SquareCompartmentDoublet(1,5e-6/p['length_scale'])

            # Set and project new initial conditions after photoactivation
            uG_n = project(G_0, V.sub(0).collapse())
            uR_n = project(R_0, V.sub(1).collapse())
            uM_n = project(M_0, V.sub(2).collapse())
            u_n = project(u0, V.sub(3).collapse())
```

```

            # Update solution vector
            assign(U_tot_n, [uG_n,uR_n,uM_n,u_n])
            uG_n, uR_n, uM_n, u_n = split(U_tot_n)

        # -----------------------------------------------------------------------------------
        # Define mechanochemical feedback
        # -----------------------------------------------------------------------------------
        # Check cellularisation parameter
        cellularisation = int(p['cellularisation'])

        # Calculate feedback based on positive strain in non-activated cells
        if cellularisation:
            feedback = p['fb']*trace_positive*non_opto_cell
        else:
            feedback = Constant(0)

        # Disable feedback if feedback strength is small
        if p['fb'] < 0.01:
            feedback = Constant(0)

        # -----------------------------------------------------------------------------------
        # Define reaction kinetics for signaling cascade
        # -----------------------------------------------------------------------------------
        # GEF activation/inactivation with mechanical feedback
        React_G = -p['lambda_decay']*uG + feedback
        # RhoA activation by GEF
        React_R = p['b']*(uG - uR)
        # Myosin activation by RhoA
        React_M = p['s']*(uR - uM)

        # -----------------------------------------------------------------------------------
        # Construct weak form of the coupled system
        # -----------------------------------------------------------------------------------
        # Reaction-diffusion equations with DG formulation
        FcG = DGWeakFormRD(uG,uG_n,vG,p['DG_N'],u,J,J_n,F,n,dx,dS,dSM,dt,cellularisation) -J*(React_G)
    *vG*dx
        FcR = DGWeakFormRD(uR,uR_n,vR,p['DR_N'],u,J,J_n,F,n,dx,dS,dSM,dt,cellularisation) -J*(React_R)
    *vR*dx
        FcM = DGWeakFormRD(uM,uM_n,vM,p['DM_N'],u,J,J_n,F,n,dx,dS,dSM,dt,cellularisation) -J*(React_M)
    *vM*dx

        # Mechanical equilibrium equation
        F_u = inner(CS,grad(vu))*dx + kN*inner(u,vu)*dx

        # Complete weak form
        FWF = FcG + FcR + FcM + F_u

        # -----------------------------------------------------------------------------------
        # Configure and solve the nonlinear system
        # -----------------------------------------------------------------------------------
        # Set solver parameters for SNES (nonlinear solver)
        snes_solver_parameters = {
            "nonlinear_solver": "snes",
            "snes_solver": {
                "linear_solver": "lu",         # Direct LU solver for linear system
                'absolute_tolerance': 1e-6,     # Convergence criteria
                'relative_tolerance': 1e-6,
                "maximum_iterations": 20,       # Limit iteration count
                "report": True,                # Print convergence info
                "error_on_nonconvergence": True
            }
        }

        # Set up nonlinear variational problem
        dFWF = derivative(FWF, U_tot, dU)   # Calculate Jacobian
```

```
        problem = NonlinearVariationalProblem(FWF, U_tot, [], J=dFWF)
        solver = NonlinearVariationalSolver(problem)
        solver.parameters.update(snes_solver_parameters)
        info(solver.parameters, False)

        # Solve the system
        (iter, converged) = solver.solve()




        # ------------------------------------------------------------------------------------
        # Prepare and save solution fields for visualization
        # ------------------------------------------------------------------------------------
        # Copy current solutions for post-processing
        U_tot_save.assign(U_tot)                # Current solution
        U_tot_save_n.assign(U_tot_n)            # Previous solution
        _uG, _uR, _uM, _u = U_tot_save.split(deepcopy=True)  # Split into components

        # ------------------------------------------------------------------------------------
        # Project solution fields onto appropriate function spaces
        # ------------------------------------------------------------------------------------
        # Vector fields (CG1 space)
        disp.assign(project(_u, V_CG1))              # Displacement field
        TractionF.assign(project(tF, V_CG1))         # Traction force

        # Molecular species concentrations (DG0 space)
        GEF.assign(project(_uG, K_DG0))              # GEF concentration
        RhoA.assign(project(_uR, K_DG0))             # RhoA concentration
        Myosin.assign(project(_uM, K_DG0))           # Myosin concentration

        # Domain markers and geometric quantities (DG0 space)
        activatedCell.assign(project(cell1, K_DG0))           # Activated cell region
        nonOptoCells.assign(project(non_opto_cell, K_DG0))    # Non-photoactivated regions
        pattern.assign(project(kN, K_DG0))                    # Micropattern
        Jacobian.assign(project(J, K_DG0))                    # Volume change
        JacobianPositive.assign(project(J_positive, K_DG0))   # Positive volume change
        feedbackPositive.assign(project(feedback, K_DG0))     # Mechanical feedback

        # Strain measures (DG0 space)
        traceGreenLagrange.assign(project(trace, K_DG0))                       # Volumetric strain
        traceGreenLagrangePositive.assign(project(trace_positive, K_DG0))      # Positive strain
        detCauchyStressPositive.assign(project(detCS_positive, K_DG0))         # Positive stress

        # Stress and strain tensors (DG0 tensor space)
        Cauchystress.assign(project(CS, W_DG0))                     # Total Cauchy stress
        Cauchystress_passive.assign(project(CS_passive, W_DG0))     # Passive stress
        activeStress.assign(project(activeStress_tensor, W_DG0))    # Active stress
        strainGreenLagrange.assign(project(eps, W_DG0))             # Strain tensor
        defGrad_save.assign(project(F, W_DG0))                      # Deformation gradient
        CauchyGreenInverse_save.assign(project(C1, W_DG0))          # Inverse Cauchy-Green tensor

        # ------------------------------------------------------------------------------------
        # Write fields to XDMF file for visualization
        # ------------------------------------------------------------------------------------
        # Vector fields
        xdmf_file.write(disp, t)                 # Displacement
        xdmf_file.write(TractionF, t)            # Traction

        # Molecular concentrations
        xdmf_file.write(GEF, t)                  # GEF
        xdmf_file.write(RhoA, t)                 # RhoA
        xdmf_file.write(Myosin, t)               # Myosin

        # Domain markers and geometric quantities
        xdmf_file.write(activatedCell, t)        # Activated cells
```

```
        xdmf_file.write(nonOptoCells, t)          # Non-photoactivated cells
        xdmf_file.write(pattern, t)               # Micropattern
        xdmf_file.write(Jacobian, t)              # Volume change
        xdmf_file.write(JacobianPositive, t)      # Positive volume change
        xdmf_file.write(feedbackPositive, t)      # Mechanical feedback

        # Strain measures
        xdmf_file.write(traceGreenLagrange, t)             # Total strain
        xdmf_file.write(traceGreenLagrangePositive, t)     # Positive strain
        xdmf_file.write(detCauchyStressPositive, t)        # Positive stress

        # Stress and strain tensors
        xdmf_file.write(Cauchystress, t)                    # Total stress
        xdmf_file.write(Cauchystress_passive, t)            # Passive stress
        xdmf_file.write(activeStress, t)                    # Active stress
        xdmf_file.write(strainGreenLagrange, t)             # Strain tensor
        xdmf_file.write(defGrad_save, t)                    # Deformation gradient
        xdmf_file.write(CauchyGreenInverse_save, t)         # Inverse Cauchy-Green

        # --------------------------------------------------------------------------------------
        # Calculate and store derived quantities
        # --------------------------------------------------------------------------------------
        # Store current time
        time_array.append(t)

        # Calculate strain energy in different regions
        dx1 = Measure('dx', domain=mesh, subdomain_data=subdomains, subdomain_id=1)
        strainEnergyLeft = calculateStrainEnergy(u, kN, dx1)      # Left cell

        dx2 = Measure('dx', domain=mesh, subdomain_data=subdomains, subdomain_id=2)
        strainEnergyRight = calculateStrainEnergy(u, kN, dx2)     # Right cell

        strainEnergyTotal = calculateStrainEnergy(u, kN, dx)      # Total system

        # Extract boundary curve for shape analysis
        boundary_curve = get_boundary_of_deformed_mesh(_u, geo_file_name)

        # Store energy and boundary data
        strainEnergyLeft_vs_time.append(strainEnergyLeft)
        strainEnergyRight_vs_time.append(strainEnergyRight)
        strainEnergyTotal_vs_time.append(strainEnergyTotal)
        boundary_curve_vs_time.append(boundary_curve)

        # --------------------------------------------------------------------------------------
        # Update time step and solution
        # --------------------------------------------------------------------------------------
        t = t + DT                      # Increment time
        U_tot_n.assign(U_tot)           # Store current solution for next step

        # --------------------------------------------------------------------------------------
        # Save simulation data to files
        # --------------------------------------------------------------------------------------
        # Create dictionary of energy data
        outputDict = {
            'time': np.array(time_array),
            'strainEnergyLeft': np.array(strainEnergyLeft_vs_time),
            'strainEnergyRight': np.array(strainEnergyRight_vs_time),
            'strainEnergyTotal': np.array(strainEnergyTotal_vs_time)
        }

        # Save energy data to CSV
        pd.DataFrame.from_dict(data=outputDict).to_csv(path_to_rawData+'strainEnergy.csv', header=True
    )

        # Save boundary curve data to NPZ file
        np.savez(path_to_rawData + 'boundary_curve.npz',
```

```python
                    boundary_curve=boundary_curve_vs_time,
                    time=np.array(time_array))

# Execute simulation if script is run directly
if __name__ == "__main__":
    simulation()
```

References

[1] Richter, T., 2017. Fluid-structure interactions: models, analysis and finite elements, volume 118. Springer.

[2] Arnold, D. N., 1982. An interior penalty finite element method with discontinuous elements. *SIAM journal on numerical analysis* 19:742–760.

[3] Wheeler, M. F., 1978. An elliptic collocation-finite element method with interior penalties. *SIAM Journal on Numerical Analysis* 15:152–161.

[4] Babuška, I., 1973. The finite element method with penalty. *Mathematics of computation* 27:221–228.

[5] Ern, A., A. F. Stephansen, and P. Zunino, 2009. A discontinuous Galerkin method with weighted averages for advection–diffusion equations with locally small and anisotropic diffusivity. *IMA Journal of Numerical Analysis* 29:235–256.

[6] Douglas, J., and T. Dupont, 1976. Interior penalty procedures for elliptic and parabolic Galerkin methods. *In* Computing methods in applied sciences, Springer, 207–216.

[7] Nitsche, J., 1971. Über ein Variationsprinzip zur Lösung von Dirichlet-Problemen bei Verwendung von Teilräumen, die keinen Randbedingungen unterworfen sind. *In* Abhandlungen aus dem mathematischen Seminar der Universität Hamburg. Springer, volume 36, 9–15.

[8] Kamps, D., J. Koch, V. O. Juma, E. Campillo-Funollet, M. Graessl, S. Banerjee, T. Mazel, X. Chen, Y. W. Wu, S. Portet, A. Madzvamuse, P. Nalbant, and L. Dehmelt, 2020. Optogenetic Tuning Reveals Rho Amplification-Dependent Dynamics of a Cell Contraction Signal Network. *Cell Reports* 33.

[9] Geuzaine, C., and J.-F. Remacle, 2008. Gmsh: a three-dimensional finite element mesh generator with built-in pre-and post-processing facilities .

[10] Staddon, M. F., E. M. Munro, and S. Banerjee, 2022. Pulsatile contractions and pattern formation in excitable actomyosin cortex. *PLoS computational biology* 18:e1009981.

[11] Citi, S., and J. Kendrick-Jones, 1987. Regulation of non-muscle myosin structure and function. *Bioessays* 7:155–159.

[12] Garcia-Mata, R., E. Boulter, and K. Burridge, 2011. The'invisible hand': regulation of RHO GTPases by RHOGDIs. *Nature reviews Molecular cell biology* 12:493–504.

[13] Seabra, M. C., 1998. Membrane association and targeting of prenylated Ras-like GTPases. *Cellular signalling* 10:167–172.

[14] Somlyo, A. P., and A. V. Somlyo, 2000. Signal transduction by G-proteins, rho-kinase and protein phosphatase to smooth muscle and non-muscle myosin II. *The Journal of physiology* 522:177–185.

[15] Lee, C.-S., C.-K. Choi, E.-Y. Shin, M. A. Schwartz, and E.-G. Kim, 2010. Myosin II directly binds and inhibits Dbl family guanine nucleotide exchange factors: a possible link to Rho family GTPases. *Journal of Cell Biology* 190:663–674.

[16] Besser, A., and U. S. Schwarz, 2007. Coupling biochemistry and mechanics in cell adhesion: A model for inhomogeneous stress fiber contraction. *New Journal of Physics* 9:1–27.

[17] Andersen, T., D. Wörthmüller, D. Probst, I. Wang, P. Moreau, V. Fitzpatrick, T. Boudou, U. Schwarz, and M. Balland, 2023. Cell size and actin architecture determine force generation in optogenetically activated cells. *Biophysical Journal* 122:684–696.

[18] Valon, L., A. Marín-Llauradó, T. Wyatt, G. Charras, and X. Trepat, 2017. Optogenetic control of cellular forces and mechanotransduction. *Nature Communications* 8.

[19] Kowalczyk, M., D. Kamps, Y. Wu, L. Dehmelt, and P. Nalbant, 2022. Monitoring the Response of Multiple Signal Network Components to Acute Chemo-Optogenetic Perturbations in Living Cells. *ChemBioChem* 23:e202100582.

[20] Valon, L., F. Etoc, A. Remorino, F. Di Pietro, X. Morin, M. Dahan, and M. Coppey, 2015. Predictive Spatiotemporal Manipulation of Signaling Perturbations Using Optogenetics. *Biophysical Journal* 109:1785–1797.

[21] Banerjee, S., and M. C. Marchetti, 2012. Contractile stresses in cohesive cell layers on finite-thickness substrates. *Physical Review Letters* 109:1–5.

[22] Beguerisse-Díaz, M., R. Desikan, and M. Barahona, 2016. Linear models of activation cascades: Analytical solutions and coarse-graining of delayed signal transduction. *Journal of the Royal Society Interface* 13.

[23] Nishikawa, M., S. R. Naganathan, F. Jülicher, and S. W. Grill, 2017. Controlling contractile instabilities in the actomyosin cortex. *Elife* 6:e19595.

[24] Michaux, J. B., F. B. Robin, W. M. McFadden, and E. M. Munro, 2018. Excitable RhoA dynamics drive pulsed contractions in the early C. elegans embryo. *Journal of Cell Biology* 217:4230–4252.

[25] Alnæs, M., J. Blechta, J. Hake, A. Johansson, B. Kehlet, A. Logg, C. Richardson, J. Ring, M. E. Rognes, and G. N. Wells, 2015. The FEniCS project version 1.5. *Archive of Numerical Software* 3.
